# Cytokines in the Germinal Center Niche

**DOI:** 10.3390/antib5010005

**Published:** 2016-02-05

**Authors:** Christoph Jandl, Cecile King

**Affiliations:** 1 Garvan Institute of Medical Research, 384 Victoria Street, Darlinghurst, Sydney, NSW 2010, Australia; c.jandl@garvan.org.au; 2 St Vincents Medical School, University of New South Wales, Sydney, NSW 2010, Australia

**Keywords:** cytokines, germinal center, T follicular helper cells, interleukin

## Abstract

Cytokines are small, secreted, glycoproteins that specifically affect the interactions and communications between cells. Cytokines are produced transiently and locally, acting in a paracrine or autocrine manner, and they are extremely potent, ligating high affinity cell surface receptors to elicit changes in gene expression and protein synthesis in the responding cell. Cytokines produced during the differentiation of T follicular helper (Tfh) cells and B cells within the germinal center (GC) niche play an important role in ensuring that the humoral immune response is robust, whilst retaining flexibility, during the generation of affinity matured antibodies. Cytokines produced by B cells, antigen presenting cells and stromal cells are important for the differentiation of Tfh cells and Tfh cell produced cytokines act both in an autocrine fashion to firm Tfh cell differentiation and in a paracrine fashion to support the differentiation of memory B cells and plasma cells. In this review, we discuss the role of cytokines during the GC reaction with a particular focus on the influence of cytokines on Tfh cells.

## 1. Introduction

The identification and analyses of soluble factors as regulators of lymphocyte functions were first studied over four decades ago [1,2]. It is now known that these soluble factors are cytokines that orchestrate the behavior of immune cells during immune responses to pathogens or following vaccination. The differentiation of antigen specific B cells into high-affinity antibody secreting plasma cells and memory B cells that provide long-term humoral immunity occurs with the help of cytokines during the germinal center (GC) reaction. GCs are temporary, specialized structures that form at the site of follicular dendritic cells (FDCs) within the B cell follicles of secondary lymphoid tissues in response to infection or immunization [3,4]. Within the dynamic environment of the GC, processes occur that are driven by antigen and cytokines, including clonal expansion, somatic hypermutation, class switch recombination and selection of high-affinity antibody producing B cells. The differentiation of affinity matured antibody producing GC B cells into plasma cells and memory B cells during the GC reaction is crucially dependent upon help from CD4^+^ T cells [3,5,6].

The CD4^+^ T cells responsible for providing help to B cells during the GC reaction are termed T follicular helper (Tfh) cells [7,8,9]. Tfh cells localize to the B cell follicle [7,8,9,10] where they provide help to B cells through cell-to-cell interactions that enable the ligation of complementary T:B cell surface expressed molecules and through Tfh cell secreted cytokines. Cytokines secreted by Tfh cells and other cells within the GC niche influence both the growth, survival and differentiation of antibody forming B cells as well as the differentiation of Tfh cells in an autocrine manner [11,12,13,14,15,16] (Table 1).

**Table 1:** **Cytokines produced within the germinal center.** Cytokines produced within the germinal center (GC) niche are listed; interleukin (IL), interferon-gamma (IFN-γ). Also listed are the cells producing these cytokines within the GC and their main function in this context, as well as the diseases in which they are implicated.

Cytokine	Main Producers	Function in the GC	Disease	Reference
**IL-21**	Tfh cells	GC B cell differentiation, affinity maturation, Tfh cell differentiation and function,	XSCID, RA, SLE, T1D	[13,14,15,16,17,18,19,20,21,22]
**IL-4**	Tfh cells	Ab class switching to IgG1, IgE	XSCID	[17,23]
**IFN-γ**	Tfh cells	Ab class switching to IgG2a	RA, SLE, T1D	[24,25]
**IL-10**	human Tfh cells, murine Tfr cells	B cell proliferation, plasma cell differentiation, Ab class switching to IgG1 and IgG3	RA, SLE, Sjogren’s syndrome, Grave’s disease	[26,27,28,29,30,31]
**IL-6**	FDCs, Plasmablasts	B cell and Tfh cell differentiation	RA, SLE	[32,33,34]
**IL-27**	unknown	Tfh cell function, IL-21 production	RA, SLE, T1D	[35,36,37]
**IL-2**	activated Th cells	Treg differentiation, negative regulator for Tfh lineage	EAE, T1D, Atherosclerosis	[38,39,40,41,42]
**IL-17**	Tfh like cells *	B cell retention in GC, Tfh cell localization to GC LZ	RA, SLE, MS, Sjogren’s syndrome	[43,44,45]

* IL-17 production by T cells happens in dysregulated GCs during autoimmunity.

Defects in cytokine signaling have been implicated in immune disorders such as X-linked severe combined immunodeficiency (XSCID) and hypogammaglobulinaemia [17,22] (Table 1), whereas the deregulation of cytokine signaling can lead to autoimmune diseases such as systemic lupus erythematosus (SLE) and rheumatoid arthritis (RA) [21,46]. The major role of cytokines in the GC reaction to T-dependent antigen has benefited from an increasing research focus over the past two decades. In this review, we provide an overview of the cytokines produced in the GC, the cells producing these cytokines, as well as their target cells and the signaling pathways involved, with a particular focus on Tfh cells.

## 2. T Follicular Helper Cells, Cytokine Producers in the Germinal Center

### 2.1. Characterization of T Follicular Helper Cells

The presence of Tfh cells within the GC reaction ensures the survival and growth/expansion of antigen-specific B cells during the process of affinity maturation [7,8,9]. In the absence of T cells, GC form, but are not maintained [47,48]. The ability of activated CD4^+^ T cells to migrate from the T cell zone into the B cell follicle is mediated by the downregulation of C-C chemokine receptor type 7 (CCR7) following TCR ligation that reduces tethering of T cells to the T cell zone where the ligands for CCR7 (the chemokines (C-C motif) ligand 19 (CCL19/ELC) and (C-C motif) ligand 21 (CCL21)) are expressed [10,49]. The reduction of CCR7 expression occurs in conjunction with increased expression of C-X-C chemokine receptor type 5 (CXCR5) [50,51]. CXCR5 is constitutively expressed on B cells where it is important for the formation of B cell follicles [52]. On CD4^+^ T cells, CXCR5 is expressed transiently upon TCR ligation [10] and is maintained at high levels on Tfh cells [7,8]. CXCR5^+^CCR7^−^CD4^+^ T cells migrate toward the ligand for CXCR5, namely chemokine (C-X-C Motif) Ligand 13 (CXCL13), which is produced by FDCs that mark the anatomical site where the GC reaction forms [49,50,51,53]. 

**Figure 1:**
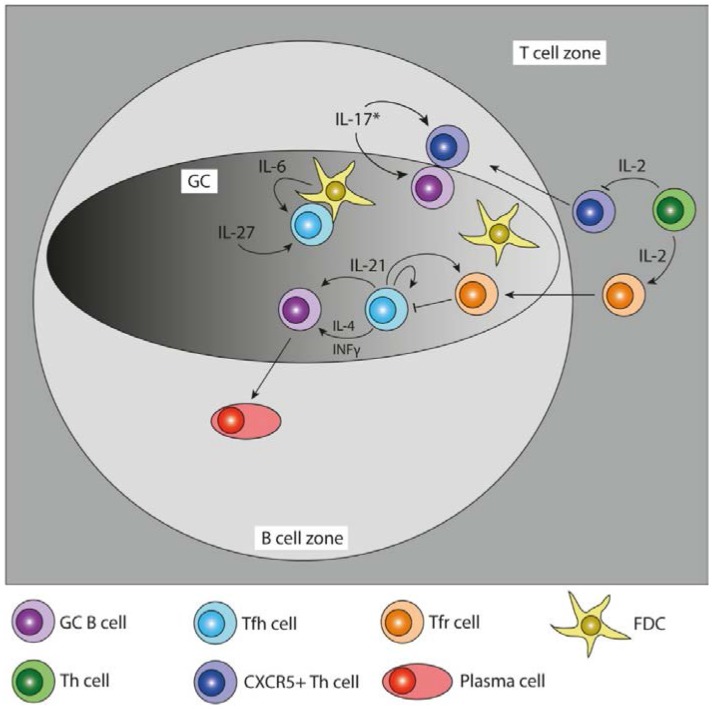
Cytokines in the germinal center (GC) reaction. Schematic diagram showing the cytokines that are important for the GC reaction and the action of these cytokines on different GC cell subsets. The relative importance of any given cytokine depends on the type of immune response during which it is expressed. Interleukin (IL), interferon-gamma (IFN-γ). *IL-17 production by T helper cells happens in dysregulated GCs during autoimmunity.

Apart from their location in the B cell follicle, Tfh cells are distinguished from other T helper cell subsets by the elevated expression of molecules that facilitate T and B cell collaboration. Direct interaction of CD40 on GC B cells and CD40L expressed by Tfh cells is indispensable for GC formation and plasma cell differentiation [54,55,56,57]. Tfh cells characteristically express high levels of ICOS, programmed cell death 1 (PD-1), the transcriptional repressor B cell lymphoma 6 (Bcl-6) as well as cytokines that influence B cell differentiation and antibody production, such as interleukin (IL)-21, IL-4 and interferon-γ (IFN-γ) [24] (Figure 1 and Table 1). In contrast to murine Tfh cells, human Tfh cells have been found to also express the cytokine IL-10, which has important functions in human B cell differentiation [58]. The engagement of ICOS with its ligand (ICOS-L) expressed on antigen presenting cells (APCs) such as B cells, leads to the production of helper cytokines such as IL-4, IL-10 [11,59] and IL-21 [13]. The Tfh cell produced cytokine IL-21 [13,18] has been shown to be a potent inducer of plasma cell differentiation in both mice and humans *in vitro* [17,60,61] and the importance of IL-21 for GC B cells is also well established [15,16].

Microarray analyses of the Tfh cell transcriptome from both mice and humans revealed a unique gene expression profile that distinguished Tfh cells from other T helper cell subsets [18,58,62,63]. Tfh cells were observed to express the highest amounts of IL-21 as well as the intracellular adaptor protein SAP (SLAM-associating protein) and the transcription factor Bcl-6 [18,62,63]. Studies using the Roquin mouse model have shown a remarkably similar transcription profile in mouse and human Tfh cells, with the most highly expressed transcripts in Tfh cells (such as *Il21*, *Cxcr5*, *CD84*, *Cxcl13*, *Bcl6* and *Pdcd1,* which encodes PD-1) detected in both organisms [62].

As the study of Tfh cells has progressed, the term Tfh cells has been used to describe CD4^+^ T cells that express CXCR5, indicating their B cell homing potential rather than localization to the GC or ability to support an affinity matured antibody response. By this definition, CXCR5hi PD1hi CD4^+^ T cells have been detected in the blood in humans and mice [7,8,64,65]. Whilst the origin of these cells remains unknown, CXCR5^+^ CD4^+^ memory T cells have been observed to migrate into the B cell follicle in response to secondary antigen challenge indicating that the maintenance of CXCR5 expression on memory CD4^+^ T cells can support immunity [66,67,68].

### 2.2. T Follicular Helper Cell Differentiation

Studies collectively demonstrate that Tfh cell differentiation is a multistage process with key checkpoints regulating the formation, migration, expansion and survival of this T helper cell subset [24]. Upon recognition of peptide-MHC class II presented by dendritic cells (DCs) in the T cell zone, CD4^+^ T cells lose expression of CCR7 and upregulate CXCR5 in a Bcl-6 dependent manner [7,8,69,70]. The activated GC Tfh “precursors” interact with cognate B cells at the T-B border, and Ag-primed T helper cells with the highest affinity for antigen [71] are thought to maintain CXCR5 expression [50,51,72]. During this “second round” of cognate interaction, the primed CD4^+^ helper cells upregulate Bcl-6 expression and become fully differentiated Tfh cells [69,70,73]. 

The differentiation, expansion and survival of Tfh cells are dependent upon signals from both DCs and B cells. Like other CD4^+^ T cell subsets, activation of Tfh cell “precursors” requires interaction with dendritic cells expressing peptide antigen in the context of MHC class II molecules. Detailed analyses of Tfh cell development revealed a broad upregulation of CXCR5, ICOS, Bcl-6, PD-1 and GL7 on CD4^+^ T cells following early (day 2–3) interaction with dendritic cells (DC) [74,75,76]. As higher TCR affinity has been associated with a preference for Tfh cell differentiation [71], prolonged interactions with DCs during the first 24h of priming leading to extended TCR and costimulatory receptor engagement as well as cytokine exposure, may bolster the Tfh differentiation program [77,78,79]. Tfh cells are able to develop in the absence of B cells, provided that adequate stimulation is available to the T cells in the form of peptide antigen-MHCII complexes on other APCs [79]. This finding may reflect the ability of B cells to act as a sufficient source of antigen for Tfh cells, but questioned whether B cells also provide any unique signals.

Although B cells are dispensable during the priming phase of CD4^+^ T cells, as well as in the initial steps of Tfh cell differentiation, they are of crucial importance for the maintenance and function of Tfh cells during the GC reaction. B cells provide an important function in supporting the expansion/survival of CD4^+^ T cells [80]. Notably, B cells support the maintenance of the Tfh cell phenotype as Bcl-6 expression and Tfh cell commitment is interrupted in the absence of T–B cell interactions [74]. Interaction with antigen-presenting B cells on the T-B border leads to the SAP-mediated secondary upregulation of Bcl-6 stabilizing the expression of CXCR5 on CD4^+^ T cells, who are then able to migrate into the GC and fully differentiate into Tfh cells [81,82,83]. Signals from ICOSL expressed on activated B cells promote persistent Tfh cell motility thus aiding Tfh migration in the B cell follicle [84].

Tfh cells differentiate under different contexts and thus must be able to assimilate a variety of input signals and this is reflected in the contribution of several transcription factors that are important for Tfh cell differentiation. T cell intrinsic Bcl-6 activity is required for Tfh cell development and GC responses to T-dependent antigen [69,70,85]. Bcl-6 can inhibit the differentiation of other (non-Tfh) CD4^+^ T cell subsets and has been shown to antagonize transcription factors important for Th1, Th2 and Th17 differentiation [69,70]. Both IL-6 and IL-21 have been shown to upregulate the expression of B*cl-6* [14,86]. Critical molecules for T–B cell interactions such as SAP, CD40L, PD-1, ICOS and CXCL13 are induced by the Tfh cell master transcription factor Bcl-6 [87]. Whilst Bcl-6 expression is high in Tfh cells, the Bcl-6 antagonist Blimp-1 is most highly expressed by other CD4^+^ T cell subsets [85,88,89]. Another transcription factor which has been shown to be important for optimal Tfh cell numbers is c-Maf, which induces IL-21 production in a ICOS-dependent manner [90] by directly binding the IL-21 promoter [91]. In addition, mice deficient in the transcription factor IRF4 (*Irf4^−/−^*) are unable to upregulate Bcl-6, CXCR5 and ICOS due to an T cell intrinsic defect and thereby fail to generate Tfh cells [92,93]. IRF4 contributes to Tfh cell differentiation, but this role is not Tfh cell specific, since *Irf4^−/−^* mice also exhibit defects in the differentiation of Th2, Th9 and Th17 cell subsets [94,95,96]. Furthermore, an CD4^+^ T cell intrinsic dependency on NF-κB-signaling for Th2 and Tfh cell induction in response to T-cell dependent Ag was found in adoptive transfer experiments with *NF-κB1^−/−^* CD4^+^ T cells [97]. NF-κB1 deficiency was shown to have detrimental effects on the production of Th2 cytokines IL-4 and IL-13 and even more notably suppressed the induction of CXCR5, whereas expression of other Tfh cell markers such as *Bcl-6, IL-21, CXCR4, OX40* mRNA as well as PD-1 protein was not impaired [97]. The importance of signaling through the trans-membrane receptor Notch for Tfh differentiation as well as IL-4 secretion by CD4^+^ T cells has been shown in mice with a CD4^+^ T cell specific deletion in both Notch1 and Notch2 (*N1N2^−/−^*) [98]. Notch-signaling is suggested to influence Tfh cell differentiation through its impact on the balance of *Bcl6* and *Blimp1* mRNA expression [98].

Although Bcl-6 is critical for the differentiation of Tfh cells, it is not exclusively expressed in Tfh cells. More recently, a Tfh specific transcription factor has been identified that drives the differentiation program of Tfh cells. Achaete-scute homologue 2 (Ascl2)—a basic helix–loop–helix (bHLH) transcription factor was selectively upregulated in Tfh cells. Ectopic expression of *Ascl2* upregulated CXCR5 (but not Bcl-6) and downregulated CCR7 expression in T cells *in vitro*, as well as both accelerating the migration of T cells to the follicles and Tfh cell development in mice [99].

### 2.3. T Follicular Regulatory Cells

Regulatory T cells (Tregs) that could suppress the humoral immune response [1,100] and CD25^+^ T helper cells that localize to the GC [101,102] have been described over the past four decades. However, it was more recently that a population of Tregs with follicular homing ability was located within the GC. CXCR5hi PD1hi FoxP3^+^ Tregs within the GC were named T follicular regulatory (Tfr) cells [31,103,104]. Foxp3^+^ Tfr cells originate from natural (thymus-derived) Treg cells (nTregs) and acquire features of Tfh cells, such as expression of CXCR5 [10,31] and high expression of PD-1 [65]; however, unlike Tfh cells, they lack expression of CD40L, IL-4 and IL-21 [31,103,104].

Tfr cells also possess an activated Treg phenotype, expressing high levels of GITR, CTLA-4 and IL-10 [31,103] (Table 1) and the transcription factor Helios, reported to be expressed by thymic nTregs but not by “inducible” Tregs (iTregs), which are induced to express FoxP3 following antigen stimulation in the periphery from FoxP3^−^ CD4^+^ T cells [105,106]. Adoptive transfer of FoxP3^+^ Treg cells into congenic recipient mice demonstrated that Tfr cells originate from nTregs and not from extrathymic CD4^+^ T cell populations such as (FoxP^−^) Tfh cells [31,103]. Tfr cells exhibit a negative regulatory function on both Tfh cells and GC B cells, as well as suppressing antigen-specific antibody production [31,107,108]. By contrast, a recent study showed that Tfr cells reduce the levels of IL-2 by utilizing IL-2 within the GC [109]. Since IL-2 inhibits Tfh cells [110], the utilization of IL-2 by Tfr cells may have a positive regulating effect on the GC reaction. Taken together, these findings suggest that Tfr cells may operate to balance the magnitude of the GC output, but aspects of their function remain incompletely understood.

## 3. Cytokine Signaling within the Germinal Center

The cytokine environment is determined by several factors including the form of immunogen and the type of secondary lymphoid tissue. Studies have provided evidence for an array of cytokines produced in the GC environment and by Tfh cells as they migrate into the GC. The earliest evidence for cytokine production within the B cell follicle and GC came from *in situ* hybridization studies. These studies showed the presence of IL-4, IL-5, IFN-γ and IL-2 to varying degrees within human low-grade lymphoma tissue [111,112] and in the lymph nodes (LN) of HIV infected individuals [113]. In mice, cytokine detection in the B cell follicle after immunization has provided variable results, including the detection of cytokine transcripts within the paracortex [114], whereas other studies have been unable to detect IL-4, IL-5 or IFN-γ in the LN of immunized mice [115]. Early studies on germinal center T cells isolated from human tonsil based upon expression of CD57 detected *IL2, IL4, IL10, IFNγ* and *TNFα* mRNA in low concentrations [116,117]. In addition to GC T cells, a strong production of *IL1β* mRNA and protein was observed in a small percentage of FDCs [117].

Improved reagents and methods to detect cytokines intracellularly by flow cytometry have facilitated the identification of cytokine producing cells (Figure 1 and Table 1). The advent of cytokine reporter mice that enable visualization of the transcription and production of cytokines in individual cells has greatly advanced our understanding of what cells produce which cytokines and when and where they do it.

### 3.1. Cytokine Reporter Mice

Recently, several groups have analyzed the relationship between IL-4-producing Tfh cells using IL-4-reporter mice [23,118,119]. These transgenic mice either express a single reporter gene marking cells that express *IL4* mRNA (IL-4-competent cells) or a combination of two reporter genes marking cells that express *IL4* mRNA as well as cells that actually secrete the IL-4 protein. The analyses of T helper cell differentiation during infection of IL-4-reporter mice confirmed previous reports showing that Tfh cells can express IL-4 [9,51,61,120] and demonstrated that the majority of IL-4 production is localized to the B cell follicle [23,118].

During *Leishmania major* infection studies of the dual IL-4 reporter mice, Tfh cells producing IL-4 could be distinguished from IL-4-producing Th2 cells by their high expression of CXCR5 and IL-21 [23]. The use of an IFN*γ*- reporter mouse crossed to the IL-4 reporter, enabled the detection of both IL-4 and IFN*γ*- producing GC T cells [23]. The isolation of T cell–B cell conjugates from the draining lymph node demonstrated that IgG1-producing B cells made contact with IL-4-producing T cells whereas IgG2a-producing B cells made contact with IFN*γ*-producing T cells [23]. IL-4-producing T cells were found conjugated to GC B cells expressing high levels of AID, with evidence of somatic hypermutation [23] demonstrating that Tfh derived cytokines direct the production of different antibody isotypes and the process of affinity maturation of antibodies in the responding B cells.

More recently, an IL-21 reporter mouse generated by introducing sequence encoding green fluorescent protein (GFP) into the *Il21* locus has enabled the identification and analyses of IL-21–GFP expressing CD4^+^CXCR5^+^PD-1^+^ Tfh cells. Following immunization with NP-KLH in aluminum hydroxide (alum), IL-21–GFP expression was restricted to a proportion of cells with a Tfh cell phenotype and IL-21–GFP^+^ Tfh cells coexpressed several cytokines, including IFN-γ, IL-2 and IL-4 [121]. IL-21–GFP^+^ Tfh cells gave rise to transferrable memory cells with plasticity, that differentiated after antigen recall into both conventional effector helper T cells and Tfh cells indicating that Tfh cells retained the flexibility to be recruited into other helper T cell subsets [121].

Further evidence for the remarkable plasticity of Th cell subsets was found when FoxP3^+^CD4^+^ T cells from FoxP3^EGFP^ mice, which express GFP under the control of the FoxP3 promoter, were transferred into T cell deficient *CD3ε^−/−^* mice [122]. In the Peyer’s patches (PP) of the small intestine, the transferred cells lost their FoxP3 expression and differentiated into Tfh cells, promoting GC formation and IgA expression [122]. These findings further supported the notion that distinct microenvironmental cues can promote the differentiation of Tfh cells from other Th cell subsets.

### 3.2. IL-21–IL6, Functional Redundancies

Differentiation of Tfh cells relies upon signal integration from several inputs including cytokines such as IL-21 and IL-6 [32,33] (Figure 1 and Table 1). Both IL-21 and IL-6 play a role in the generation of a GC reaction of optimal magnitude. Studies have shown that mice made genetically deficient in either the receptor for IL-21 (*Il21r^−/−^* mice), IL-21 (*Il21^−/−^* mice) or IL-6 (*Il6^−/−^* mice) have GC reactions of reduced magnitude [13,14,123]. *Il21^−/−^*, *Il21r^−/−^* and *Il6^−/−^* mice have also been reported to exhibit decreased Tfh cell numbers after immunization with T-dependent antigen [13,14]. However, the influence of IL-21 on Tfh cells has been context dependent; influenced by the immunogen/adjuvant, whether the response is primary or secondary and also influenced by the surface markers used to define Tfh cells [13,15,16,68,124]. Importantly, IL-6 and IL-21 appear to have redundant functions for Tfh cell differentiation. Whilst the absence of either IL-6 or IL-21 alone only has a modest impact, [73], eliminating signals from both cytokines severely diminished the percentages of Tfh cells following infection [32,33]. 

IL-6 expressed by plasmablasts can act as a signal driving Tfh cell formation in humans [125], and stromal cells in the B cell follicle such as FDCs have also been identified as a potential source of IL-6 [123,126] (Figure 1). Although *Il21r^−/−^* mice did not show a difference in numbers of either mature or immature B cells in comparison to wild type mice [17], lower levels of IgG1 and higher levels of IgE could be detected in naive *Il21r^−/−^* mice compared with wild-type mice [17,127,128].

Both IL-6 and IL-21 signal through the JAK-STAT pathway inducing activation of janus kinase (JAK)1 as well as JAK3, which in turn leads to the activation of *signal transducer and activator of transcription (*STAT)1 and STAT3 [86,129,130,131]. STATs are DNA-binding transcription factors that influence gene transcription by binding DNA target sites as homodimers or heterodimers and can form transcriptional complexes with non-STAT transcription factors [132]. The observation that both IL-21 and IL-6 activate STAT1 and STAT3 upon binding to their respective receptors may underpin the observed functional overlap between these two cytokines in Tfh cell differentiation [32,33,86,127,133].

STAT1 activation by IL-6 signaling has been shown to be critical for Bcl-6 induction and subsequent differentiation of Tfh cells [86]. Whether IL-21 mediated STAT1 signaling is important for the Tfh differentiation program remains unknown. The activation of STAT3 is required for the production of IL-21 by CD4^+^ T cells from mice and humans after stimulation with IL-6, IL-21, IL-23 and IL-27 [14,36,134] (Table 1). Additionally, it was shown that STAT1 and STAT3 cooperate in promoting Tfh differentiation in response to IL-6 [86]. The importance of IL-21 mediated STAT3 signaling for B cells has been demonstrated in human B cells *in vitro* [129] and in murine plasma cells *in vivo* [135].

Although STAT3 can bind the *Bcl6* gene and upregulate its expression, STAT3 is also a strong inducer of Blimp-1 expression in B cells [92,129,136]. By contrast, constitutive expression of Blimp-1 suppresses Bcl-6 expression in CD4^+^ T cells, leading to defects in Tfh cell differentiation [85]. The deletion of *Blimp-1* from CD4^+^ T cells leads to augmented Tfh cell formation in response to viral infection as this transcription factor directly suppresses Bcl-6 expression [74,85].

Expression of Bcl-6 is upregulated by IL-6 as well as IL-21 in an autocrine manner in T cells [69,70,137]. Furthermore, it has been shown that IL-6 is not only able to function in place of IL-21 but it can also induce the production of IL-21 and influence Tfh cell differentiation in this way [138]. The roles of IL-6 and IL-21 on both B cells and Tfh cells during the GC reaction may reflect the dual ability of IL-6 and IL-21 to upregulate critical GC transcription factors such as Bcl-6 in both B and T cells. Similar to IL-6 and IL-21, IL-27 signaling involves the activation of Jak1, STAT1, and STAT3 [139]. IL-27 has also been shown to be important for the function of Tfh cells and for affinity matured antibody responses [36] (Figure 1 and Table 1). IL-27 signaling to T cells results in the production of IL-21 and improves Tfh cell survival in a STAT3-dependent manner [36]. Recent studies have built on our knowledge base to explain the important contribution of both IL-6 and IL-21 to Tfh cell differentiation and the GC reaction. However, important questions remain including the timing of the production of IL-6, IL-21 and IL-27 during Tfh differentiation and the GC reaction in different contexts of infection and immunisation.

### 3.3. IL-21–IL-2, Antagonizing Functions

The evolution of the genes that encode the known array of cytokines and receptor complexes involved multiple duplications from a smaller set of genes, followed by the divergence of sequence and product function [140]. The cytokines IL-2 and IL-21 are encoded on adjacent genes on chromosome 3 in mice and chromosome 4 in humans and are thought to have arisen by gene duplication. Both IL-2 and IL-21 are secreted by CD4^+^ T cells, but studies show that they exert opposing functions in the differentiation and function of CD4^+^ T cell subsets [13,141,142,143,144]. Whereas IL-2 is an important survival factor for forkhead family transcription factor (FoxP3) expressing Tregs that are vital for the regulation of immune responses in both mice and humans [141,142,145,146], IL-21 can inhibit the expansion and function of FoxP3^+^ Tregs [143,147,148]. IL-21 is one of the most highly expressed genes in *Il2^−/−^* mice [149], indicating that regulation of IL-21 or IL-21-producing Th cells is an important role of Tregs.

Recent studies have contributed significantly to our appreciation of how the interplay between IL-2 and IL-21 can shape the outcome of antibody responses. Tregs expand to a greater extent in *Il21^−/−^* mice than in WT mice after immunization and treatment with anti-CD28 mAbs [13]. IL-21 signaling can inhibit the expansion of Tregs both *in vitro* [143] as well as after infection with LCMV [144]. The negative effect of IL-21 on Treg homeostasis and suppressor function has been proposed to be achieved by IL-21 mediated down regulation of FoxP3 expression on Tregs and by inhibiting IL-2 production from activated T cells (T conventional cells), thereby limiting its availability as a growth and survival factor for Tregs [143,147,150,151,152].

Tregs are dependent upon IL-2 for their growth and survival and the size of the Treg population is directly correlated with the amount of bioavailable IL-2 [38,39] (Table 1). By contrast, IL-2 has an inhibitory effect on Tfh cell differentiation, thereby counteracting one of the most important functions of IL-21 [110,153,154] (Table 1). IL-2 induces Blimp-1 expression in CD4^+^ T cells, and administration of IL-2 in an influenza virus infection model leads to reduced Tfh cell numbers as well as less antibody production, thereby suggesting an antagonistic role for IL-2 and IL-21 on Tfh cells [110]. As IL-2 mainly signals through STAT5, its limiting effect on Tfh cell formation has been suggested to operate through the suppression of Bcl-6 through STAT5 mediated upregulation of Blimp-1 [42,153]. One recent study was also able to show increased Tfr cell numbers as well as decreased Tfh cell numbers in the absence of IL-21 signaling in lupus-prone BXD2 mice [155], further highlighting the important role of IL-21 on CD4^+^ T cells in the GC reaction. Two mechanisms for the negative effect of IL-2 on Tfh cells have been proposed. Firstly, IL-2 mediated activation of STAT5 leads to activation of the transcriptional repressor Blimp-1, which, in turn, leads to the repression of the transcription factor Bcl-6, thereby negatively regulating Tfh cell differentiation and proliferation [153,154]. Secondly, STAT5, activated by IL-2, is able to displace STAT3 bound to the Bcl-6 promoter, adversely influencing Tfh cell formation in this manner [42].

### 3.4. IL-21–IL-4, Collaborative Functions

As discussed earlier, while IL-21 can be considered the signature cytokine of the Tfh cell lineage, it has been demonstrated that these cells also secrete other cytokines, such as IL-4 [23,121]. The expression of IL-4 by Tfh cells is regulated by the transcription factor Batf [156]. Together with IRF4 and in concert with STAT3 and STAT6, Batf binds to the *CNS2* region of the *Il4* locus and thereby promotes IL-4 production in Tfh cells [156]. Apart from its role in IL-4 production, Batf has also been shown to control expression of the transcription factors Bcl-6 and c-Maf leading to impaired Th17 and Tfh cell development in *Batf^−/−^* mice [157,158]. Although the effect of IL-21 on the differentiation of naïve human B cells into antibody secreting cells could be decreased by IL-4 through its antagonizing effect on IL-21 induced Blimp-1 expression [60,61], the stimulation of human B cells in culture with both IL-4 and IL-21 resulted in increased production of IgE [159,160]. The ability of IL-21 to promote IgE production from human B cells *in vitro* stands in contrast to the observation of elevated IgE in humans with loss of function mutations in *IL21R* and in both *Il21r^−/−^* and *Il21^−/−^* mice [16,17,161].

Paradoxically, despite reduced IL-4 detected in sera and reduced production of IgG1 after infection, *Il21r−/−* mice harbor increased amounts of the antibody isotype IgE [17]. The mechanism for the inhibitory effect of IL-21 on IgE has been explored in several studies. IL-21 has been shown to down-regulate IgE production from IL-4-stimulated B cells through the inhibition of germ line C(epsilon) transcription [162]. In addition, the transcriptional repressor Bcl-6 negatively regulates the Iε promoter [163] and, in this manner, IL-21 may negatively regulate IgE production by increasing the expression of Bcl-6 in B cells [15]. More recently, *Il21r^−/−^* B cells and T cells were observed to exhibit reduced surface expression of IL-4Ra, which was accompanied by reduced production of soluble IL-4Ra [124], sIL-4Ra is known to bind IL-4 and reduce IgE levels in mice, but whether reduced sIL-4Ra contributes to the increased IgE observed in *Il21r^−/−^* mice remains unknown [124].

The critical dual role of IL-4 and IL-21 in antibody production was highlighted in mice lacking both IL-4 and IL-21R (*Il4^−/−^Il21r^−/−^*) that exhibited pan-hypogammaglobulinaemia [17]. In addition to the lack of IgG isotypes, the double knock-out mice lacked the increased amounts of IgE observed in *Il21r^−/−^* mice, confirming the IL-4 dependency of the elevated IgE in the absence of IL-21:IL-21R signaling [17]. The B cell phenotype shown by *Il4^−/−^Il21r^−/−^* mice was similar to human patients with XSCID, leading to the hypothesis that this disease could be caused by the combined loss of signaling mediated by these two cytokines [17].

### 3.5. Interferon-γ, A Non-Canonical Cytokine Expressed by Tfh Cells

Expression of IFN-γ by Th cells is important for IgG2a class switching of murine B cells. Low expression of IFN-γ by CXCR5hi Tfh cells could be detected in human tonsils [70,164], as well as mice immunized with sheep red blood cells [23,85,121]. By contrast, in comparison to CD4^+^ T cells that express intermediate levels of CXCR5, a substantial increase of IFN-γ production by Tfh cells has been observed during viral infection [165]. A close relationship between Tfh cells and IFN-γ has been recently demonstrated in lupus-prone *sanroque* mice. Elevated expression of IFN-γ present in the GC of *sanroque* mice leads to increased numbers of pathogenic Tfh cells and autoimmune antibody responses, suggesting a role for IFN-γ for Tfh cell formation [166]. Developing Tfh cells have been shown to repress IFN-γ expression through the upregulation of the transcriptional repressor Bcl-6 [70], which is consistent with the quantitatively low expression of GATA3 and T-bet in Tfh cells in comparison to other CD4^+^ T cell subsets [23,85,165], supporting the idea that low levels of IFN-γ expressed by Tfh cells are sufficient for IgG2a class switching [24].

### 3.6. IL-21–IL-10, Differential Functions in Humans and Mice

Interleukin-10 has been shown to have multiple effects on various types of immune cells, most notably it functions as a survival, proliferation and differentiation factor for B cells [26,27,167]. Before IL-21 was described, IL-10 had been considered the most potent inducer of human plasma cell differentiation [28,29,61]. IL-10 promotes immunoglobulin class switching to IgG1 and IgG3, and is a strong inducer of immunoglobulin secretion [28]. The production of IL-10 by T cells follows stimulation through the T cell receptor (TCR), CD28 and ICOS [29,168], and there is a direct relationship between ICOS expression by CD4^+^ T cells and the amount of IL-10 produced [168,169].

Whilst IL-10 is a strong inducer of plasma cell differentiation in humans, it is associated with both immunosuppressive [170] and proinflammatory effects in mice [171]. By contrast to the effect of IL-10 on B cells, IL-10 has a negative effect on Tfh cell differentiation in mice. At least two different mechanisms have been demonstrated: First, B cells co-cultured with Tfh cells deficient for the IL-10R subunit β (*Il10rb*^−/−^) showed increased antibody responses *in vitro* [172], demonstrating Tfh cell intrinsic inhibitory effect of IL-10. Secondly, the inability of DCs in *Il10rb*^−/−^ mice to respond to IL-10 resulted in higher levels of IL-6, IL-12 and IL-23 in these mice, which resulted in higher Tfh cell numbers [172]. Additionally, the expression of IL-17 and IL-21 was increased in the IL-10R deficient mice, which suggests a regulatory role of IL-10 on both number and function of Tfh cells in mice [172]. The fact that IL-10 negatively regulates Tfh cells both in normal and autoimmune settings [172,173] might explain one of the mechanisms of Tfr cell mediated regulation of Tfh cells, since Tfr cells have been shown to express *Il10* mRNA [31] (Figure 1). How exactly IL-10 negatively regulates Tfh cells in mice is still unknown, but IL-10 mediated induction of Blimp-1 has been suggested as one possible mechanism [174]. An important source of IL-10 may be Tregs under certain settings, but the finding that *Il2^−/−^* mice that are deficient in IL-2-dependent FoxP3^+^ Tregs have significantly greater amounts of IL-10 in serum compared with WT mice indicates that IL-10 is produced by a variety of cells and further illustrates the complex role of IL-10 during immune responses [149].

### 3.7. IL-21–IL-17, Complementing Functions in Autoimmune Disease

A potent role for IL-17 in the production of self-reactive Ab and the formation of GCs in autoimmune disease has been established [43,175,176]. Although IL-17 is not produced by human tonsilar Tfh cells or murine Tfh cells induced by immunization [70], elevated levels of this cytokine in systemic autoimmunity prone BXD2 mice leads to spontaneous formation of GCs [43]. The IL-17 receptor (IL-17RA) was shown to be highly expressed by both Tfh cells and GC B cells in BXD2 mice [43,45] (Figure 1 and Table 1). The aberrant production of IL-17 in the context of the GC leads to the upregulated expression of the genes *Rsg13* and *Rsg16* in B cells of BXD2 mice, both of which have been shown to inhibit CXCR4-CXCL12 signaling and therefore B cell migration [177,178]. A more recent study suggested that IL-17 signaling is of high importance for optimal localization of Tfh cells into the light zone (LZ) of the GC in BXD2 mice [45]. Although IL-17 is not needed for Tfh cell development *per se*, it complements the effects of IL-21 since the loss of either cytokine abrogates development of autoimmune GCs in BXD2 mice [45]. T helper 17 (Th17) cells producing IL-17 are highly proinflammatory effector T cells associated with a number of autoimmune diseases [179], and they can provide help to B cells and mediate class switching to IgG1 and IgG2b as well as to a lesser extent to IgG2a and IgG3 [180]. Th17 cells have also been shown to mediate GC formation [180] and even acquire a Tfh-like phenotype in the environment of the payers’ patches of the small intestine [181]. Further evidence for IL-17 producing Tfh-like cells was found in *RORγt^−/−^* mice [182]. However, whether these cells constitute a genuine new subpopulation of Tfh cells or provide another example of the remarkable plasticity between Th cell subsets within distinct microenvironments remains unknown.

## 4. Roles for Germinal Center Cytokine Signaling in Disease

Not only are GC reactions important for humoral immune responses against pathogens and long lasting immunological memory, but defects in regulating GC cytokines can also lead to autoimmune disease or immune deficiencies. Mutations in the in the *IL2RG* gene, encoding the γc through which signaling for the cytokines IL-2, IL-4, IL-7, IL-9, IL-15 and IL-21 occurs, cause the disease XSCID in humans [19,131,183]. This disease is characterized by the absence of T cells and NK cells, and although B cell numbers in patients with XSCID are normal, the B cells seem to be unable to perform their proper function [184]. IL-21 imparts both autocrine and paracrine effects on lymphocytes influencing survival and differentiation [185,186,187] and contributes to the development of inflammatory and autoimmune diseases [186] (Table 1). In humans, loss-of-function mutations in *IL21* or *IL21R* cause a primary immunodeficiency syndrome. These patients exhibit defects in antibody production, T cell and NK cell functions and an increased susceptibility to chronic infections and gastrointestinal inflammation [161,188,189].

Genome-wide association studies have identified the locus encoding *Il21* and *Il2* as a risk factor for autoimmune diseases such as systemic lupus erythematosus (SLE), type 1 diabetes, inflammatory bowel disease, coeliac disease, psoriasis and psoriatic arthritis [20,21,190,191,192]. Dysregulated Tfh cells, including increased Tfh cell numbers and production of IL-21, have been implicated in a number of autoimmune diseases [20,21,190,191,192,193].

The BXSB-*Yaa* mouse model for SLE is characterized by an accumulation of IL-21 expressing Tfh cells [22,194]. These mice develop severe SLE symptoms such as spontaneous GC formation, increased autoantibody production, hypergammaglobulinemia and glomerulonephritis [136,195]. Crossing the BXSB-*Yaa* mice with a *Il21r^−/−^* mouse line alleviated disease symptoms, improved mortality and resulted in a significant reduction in the production of autoreactive antibodies [22,194]. The benefit of the therapeutic approach of blocking IL-21 signaling in autoimmune settings has been demonstrated in several studies [22,196,197,198]. In addition to IL-21, therapeutic targeting of other GC cytokines has a beneficial effect on mouse models of Lupus. Blockade of the interaction of IL-4 with its receptor by antibody treatment or of its downstream signaling by inactivation of the Stat6 gene ameliorates glomerulosclerosis as well as delaying the development of end-stage renal disease [199,200].

Elevated levels of IL-21 protein and mRNA have been detected in human patients suffering from RA, a chronic autoimmune disease characterized by inflammation of synovial joints and the expression of autoreactive antibodies leading to the destruction of bone and cartilage [196]. Increased frequencies of circulating Tfh cells have been detected in RA patients and clinical studies showed that the elevated levels of IL-21 in these patients decreases following treatment, which correlates with improvement of disease activity [46,201,202]. Additionally, observations made in mouse models of RA suggest an essential role for IL-21 in disease development [203]. Similar to IL-21, patients with RA have high synovial levels of IL-6 [34] and therapeutically targeting IL-6 receptors has been shown to be beneficial for many RA patients [204].

Taken together, these studies demonstrate that cytokines have emerged as important prognostic factors in human disease, and the therapeutic targeting of cytokines produced within the GC niche has proved beneficial in the treatment of antibody mediated autoimmune diseases.

## 5. Conclusions

In order to respond to a variety of pathogens, the immune response must be agile and this is reflected in the context dependent differences observed in the GC reaction. Cytokines operate across short (cell–cell) distances, acting in an autocrine and/or paracrine manner. The cytokine milieu in the GC is dependent upon the type of secondary lymphoid tissue, the cell types that form the GC locus and those that migrate there to participate in the GC reaction. Tfh cells begin their process of differentiation in the T cell zone of secondary lymphoid tissues, where cytokines produced by T cells and antigen-presenting cells are prominent. As Tfh cells migrate into the B cell follicle and GC, they encounter cytokines produced by other Tfh cells, B cells and FDCs. Antigen-specific B cells, in turn, begin the process of class switch in response to T cell produced cytokines prior to their migration into the GC, where they encounter cytokines produced by Tfh cells and FDCs that influence GC B cell proliferation, survival and differentiation.

Recent advances in Tfh cell biology have focused our attention on cytokines in the GC niche. These advances have been made possible by state of the art techniques such as intravital microscopy and cytokine-reporter mice that enable the detection and tracking of small numbers of cells during the GC reaction. However, despite such advances, many questions remain unanswered. They include the kinetics of cytokine production in the context of Tfh cell and GC B cell differentiation. GC cytokines use both overlapping and discrete signaling pathways to elicit their effects on responding cells. How multiple cytokines signaling through common pathways influence cell differentiation remains incompletely understood. The relative contributions of cytokine signaling components to the behavior and differentiation of cells that participate in the GC reaction, including the roles of individual STAT proteins in both homodimeric and heterodimeric forms, remains an important unanswered question.
