# Correction: Cytokines in the Germinal Center Niche *Antibodies* 2016, *5*(1), 5

**DOI:** 10.3390/antib5020010

**Published:** 2016-04-27

**Authors:** Christoph Jandl, Cecile King

**Affiliations:** 1 Garvan Institute of Medical Research, 384 Victoria Street, Darlinghurst, Sydney, NSW 2010, Australia; 2 St Vincents Medical School, UNSW, NSW 2010, Australia; c.jandl@garvan.org.au

The following corrections should be made to the references and bibliography of the published paper [1]:

Reference number [26] should be replaced by the number [31] on page 5, lines 21, 23, 24, 26, 30, 32, as well as on page 9, line 40.

Reference number [35] should be replaced by the number [36] on page 7, lines 20, 35 and 36.

On page 9, line 23, reference number [27] should be replaced by the number [26].

Also on page 9, line 23, reference number [28] should be replaced by the number [27]. 

Reference number [29] should be replaced by the number [28] on page 9, lines 25 and 26.

Also on page 9, reference number [30] should be replaced by the number [29] on lines 25 and 27.

Reference number [44] should be replaced by the number [45] on page 10, lines 6, 10, and 12. 

Lastly, the bibliography should be updated to read:

1. Gershon, R.K.; Kondo, K. Cell interactions in the induction of tolerance: The role of thymic lymphocytes. *Immunology* **1970**, *18*, 723–737.

2. Mier, J.W.; Gallo, R.C. Purification and some characteristics of human T-cell growth factor from phytohemagglutinin-stimulated lymphocyte-conditioned media. *Proc. Natl. Acad. Sci. USA* **1980**, *77*, 6134–6138.

3. MacLennan, I.C.M. Germinal Centers. *Annu. Rev. Immunol.* **1994**, *12*, 117–139.

4. Liu, Y.J.; Malisan, F.; de Bouteiller, O.; Guret, C.; Lebecque, S.; Banchereau, J.; Mills, F.C.; Max, E.E.; Martinez-Valdez, H. Within germinal centers, isotype switching of immunoglobulin genes occurs after the onset of somatic mutation. *Immunity* **1996**, *4*, 241–250.

5. Claman, H.N.; Chaperon, E.A.; Triplett, R.F. Thymus-marrow cell combinations. Synergism in antibody production. *Proc. Soc. Exp. Biol. Med. Soc. Exp. Biol. Med. N. Y. N.* **1966**, *122*, 1167–1171.

6. Miller, J.F.; Mitchell, G.F. Cell to cell interaction in the immune response. I. Hemolysin-forming cells in neonatally thymectomized mice reconstituted with thymus or thoracic duct lymphocytes. *J. Exp. Med.* **1968**, *128*, 801–820.

7. Breitfeld, D.; Ohl, L.; Kremmer, E.; Ellwart, J.; Sallusto, F.; Lipp, M.; Förster, R. Follicular B helper T cells express CXC chemokine receptor 5, localize to B cell follicles, and support immunoglobulin production. *J. Exp. Med.* **2000**, *192*, 1545–1552.

8. Schaerli, P.; Willimann, K.; Lang, A.B.; Lipp, M.; Loetscher, P.; Moser, B. CXC chemokine receptor 5 expression defines follicular homing T cells with B cell helper function. *J. Exp. Med.* **2000**, *192*, 1553–1562.

9. Kim, C.H.; Rott, L.S.; Clark-Lewis, I.; Campbell, D.J.; Wu, L.; Butcher, E.C. Subspecialization of CXCR5+ T cells: B helper activity is focused in a germinal center-localized subset of CXCR5+ T cells. *J. Exp. Med.* **2001**, *193*, 1373–1381.

10. Ansel, K.M.; McHeyzer-Williams, L.J.; Ngo, V.N.; McHeyzer-Williams, M.G.; Cyster, J.G. *In vivo*-activated CD4 T cells upregulate CXC chemokine receptor 5 and reprogram their response to lymphoid chemokines. *J. Exp. Med.* **1999**, *190*, 1123–1134.

11. Hutloff, A.; Dittrich, A.M.; Beier, K.C.; Eljaschewitsch, B.; Kraft, R.; Anagnostopoulos, I.; Kroczek, R.A. ICOS is an inducible T-cell co-stimulator structurally and functionally related to CD28. *Nature* **1999**, *397*, 263–266.

12. van Kooten, C.; Banchereau, J. CD40-CD40 ligand. *J. Leukoc. Biol.* **2000**, *67*, 2–17.

13. Vogelzang, A.; McGuire, H.M.; Yu, D.; Sprent, J.; Mackay, C.R.; King, C. A fundamental role for interleukin-21 in the generation of T follicular helper cells. *Immunity* **2008**, *29*, 127–137.

14. Nurieva, R.I.; Chung, Y.; Hwang, D.; Yang, X.O.; Kang, H.S.; Ma, L.; Wang, Y.; Watowich, S.S.; Jetten, A.M.; Tian, Q.; Dong, C. Generation of T follicular helper cells is mediated by interleukin-21 but independent of T helper 1, 2, or 17 cell lineages. *Immunity* **2008**, *29*, 138–149.

15. Linterman, M.A.; Beaton, L.; Yu, D.; Ramiscal, R.R.; Srivastava, M.; Hogan, J.J.; Verma, N.K.; Smyth, M.J.; Rigby, R.J.; Vinuesa, C.G. IL-21 acts directly on B cells to regulate Bcl-6 expression and germinal center responses. *J. Exp. Med.* **2010**, *207*, 353–363.

16. Zotos, D.; Coquet, J.M.; Zhang, Y.; Light, A.; D’Costa, K.; Kallies, A.; Corcoran, L.M.; Godfrey, D.I.; Toellner, K.-M.; Smyth, M.J.; *et al*. IL-21 regulates germinal center B cell differentiation and proliferation through a B cell–intrinsic mechanism. *J. Exp. Med.* **2010**, *207*, 365–378.

17. Ozaki, K.; Spolski, R.; Feng, C.G.; Qi, C.-F.; Cheng, J.; Sher, A.; Morse, H.C., 3rd; Liu, C.; Schwartzberg, P.L.; Leonard, W.J. A critical role for IL-21 in regulating immunoglobulin production. *Science* **2002**, *298*, 1630–1634.

18. Chtanova, T.; Tangye, S.G.; Newton, R.; Frank, N.; Hodge, M.R.; Rolph, M.S.; Mackay, C.R. T follicular helper cells express a distinctive transcriptional profile, reflecting their role as non-Th1/Th2 effector cells that provide help for B cells. *J. Immunol. Baltim. Md 1950* **2004**, *173*, 68–78.

19. Leonard, W.J. Cytokines and immunodeficiency diseases. *Nat. Rev. Immunol.* **2001**, *1*, 200–208.

20. Cooper, J.D.; Smyth, D.J.; Smiles, A.M.; Plagnol, V.; Walker, N.M.; Allen, J.E.; Downes, K.; Barrett, J.C.; Healy, B.C.; Mychaleckyj, J.C.; *et al.* Meta-analysis of genome-wide association study data identifies additional type 1 diabetes risk loci. *Nat. Genet.* **2008**, *40*, 1399–1401.

21. Hughes, T.; Kim-Howard, X.; Kelly, J.A.; Kaufman, K.M.; Langefeld, C.D.; Ziegler, J.; Sanchez, E.; Kimberly, R.P.; Edberg, J.C.; Ramsey-Goldman, R.; *et al*. Fine-mapping and transethnic genotyping establish IL2/IL21 genetic association with lupus and localize this genetic effect to IL21. *Arthritis Rheum.* **2011**, *63*, 1689–1697.

22. Bubier, J.A.; Sproule, T.J.; Foreman, O.; Spolski, R.; Shaffer, D.J.; Morse, H.C.; Leonard, W.J.; Roopenian, D.C. A critical role for IL-21 receptor signaling in the pathogenesis of systemic lupus erythematosus in BXSB-Yaa mice. *Proc. Natl. Acad. Sci. USA* **2009**, *106*, 1518–1523.

23. Reinhardt, R.L.; Liang, H.-E.; Locksley, R.M. Cytokine-secreting follicular T cells shape the antibody repertoire. *Nat. Immunol.* **2009**, *10*, 385–393.

24. Crotty, S. Follicular helper CD4 T cells (TFH). *Annu. Rev. Immunol.* **2011**, *29*, 621–663.

25. Arellano, G.; Ottum, P.A.; Reyes, L.I.; Burgos, P.I.; Naves, R. Stage-Specific Role of Interferon-Gamma in Experimental Autoimmune Encephalomyelitis and Multiple Sclerosis. *Front. Immunol.* **2015**, *6*, 492.

26. Go, N.F.; Castle, B.E.; Barrett, R.; Kastelein, R.; Dang, W.; Mosmann, T.R.; Moore, K.W.; Howard, M. Interleukin 10, a novel B cell stimulatory factor: Unresponsiveness of X chromosome-linked immunodeficiency B cells. *J. Exp. Med.* **1990**, *172*, 1625–1631.

27. Wakkach, A.; Cottrez, F.; Groux, H. Can interleukin-10 be used as a true immunoregulatory cytokine? *Eur. Cytokine Netw.* **2000**, *11*, 153–160.

28. Banchereau, J.; Rousset, F. Human B lymphocytes: Phenotype, proliferation, and differentiation. *Adv. Immunol.* **1992**, *52*, 125–262.

29. Rousset, F.; Garcia, E.; Defrance, T.; Péronne, C.; Vezzio, N.; Hsu, D.H.; Kastelein, R.; Moore, K.W.; Banchereau, J. Interleukin 10 is a potent growth and differentiation factor for activated human B lymphocytes. *Proc. Natl. Acad. Sci. USA* **1992**, *89*, 1890–1893.

30. Lalani, I.; Bhol, K.; Ahmed, A.R. Interleukin-10: Biology, role in inflammation and autoimmunity. *Ann. Allergy Asthma Immunol. Off. Publ. Am. Coll. Allergy Asthma Immunol.* **1997**, *79*, 469–483.

31. Linterman, M.A.; Pierson, W.; Lee, S.K.; Kallies, A.; Kawamoto, S.; Rayner, T.F.; Srivastava, M.; Divekar, D.P.; Beaton, L.; Hogan, J.J.; *et al*. Foxp3+ follicular regulatory T cells control the germinal center response. *Nat. Med.* **2011**, *17*, 975–982.

32. Eto, D.; Lao, C.; DiToro, D.; Barnett, B.; Escobar, T.C.; Kageyama, R.; Yusuf, I.; Crotty, S. IL-21 and IL-6 Are Critical for Different Aspects of B Cell Immunity and Redundantly Induce Optimal Follicular Helper CD4 T Cell (Tfh) Differentiation. *PLoS ONE* **2011**, *6*, 3.

33. Karnowski, A.; Chevrier, S.; Belz, G.T.; Mount, A.; Emslie, D.; D’Costa, K.; Tarlinton, D.M.; Kallies, A.; Corcoran, L.M. B and T cells collaborate in antiviral responses via IL-6, IL-21, and transcriptional activator and coactivator, Oct2 and OBF-1. *J. Exp. Med.* **2012**, *209*, 2049–2064.

34. Hirano, T. Interleukin 6 and its Receptor: Ten Years Later. *Int. Rev. Immunol.* **1998**, *16*, 249–284.

35. Meka, R.R.; Venkatesha, S.H.; Dudics, S.; Acharya, B.; Moudgil, K.D. IL-27-induced modulation of autoimmunity and its therapeutic potential. *Autoimmun. Rev.* **2015**, *14*, 1131–1141.

36. Batten, M.; Ramamoorthi, N.; Kljavin, N.M.; Ma, C.S.; Cox, J.H.; Dengler, H.S.; Danilenko, D.M.; Caplazi, P.; Wong, M.; Fulcher, D.A.; *et al*. IL-27 supports germinal center function by enhancing IL-21 production and the function of T follicular helper cells. *J. Exp. Med.* **2010**, *207*, 2895–2906.

37. Batten, M.; Li, J.; Yi, S.; Kljavin, N.M.; Danilenko, D.M.; Lucas, S.; Lee, J.; de Sauvage, F.J.; Ghilardi, N. Interleukin 27 limits autoimmune encephalomyelitis by suppressing the development of interleukin 17-producing T cells. *Nat. Immunol.* **2006**, *7*, 929–936.

38. Boyman, O.; Kovar, M.; Rubinstein, M.P.; Surh, C.D.; Sprent, J. Selective stimulation of T cell subsets with antibody-cytokine immune complexes. *Science* **2006**, *311*, 1924–1927.

39. Webster, K.E.; Walters, S.; Kohler, R.E.; Mrkvan, T.; Boyman, O.; Surh, C.D.; Grey, S.T.; Sprent, J. *In vivo* expansion of T reg cells with IL-2-mAb complexes: Induction of resistance to EAE and long-term acceptance of islet allografts without immunosuppression. *J. Exp. Med.* **2009**, *206*, 751–760.

40. Setoguchi, R.; Hori, S.; Takahashi, T.; Sakaguchi, S. Homeostatic maintenance of natural Foxp3(+) CD25(+) CD4(+) regulatory T cells by interleukin (IL)-2 and induction of autoimmune disease by IL-2 neutralization. *J. Exp. Med.* **2005**, *201*, 723–735.

41. Sharma, R.; Fu, S.M.; Ju, S.-T. IL-2: A two-faced master regulator of autoimmunity. *J. Autoimmun.* **2011**, *36*, 91–97.

42. Oestreich, K.J.; Mohn, S.E.; Weinmann, A.S. Molecular mechanisms that control the expression and activity of Bcl-6 in TH1 cells to regulate flexibility with a TFH-like gene profile. *Nat. Immunol.* **2012**, *13*, 405–411.

43. Hsu, H.-C.; Yang, P.; Wang, J.; Wu, Q.; Myers, R.; Chen, J.; Yi, J.; Guentert, T.; Tousson, A.; Stanus, A.L.; *et al*. Interleukin 17-producing T helper cells and interleukin 17 orchestrate autoreactive germinal center development in autoimmune BXD2 mice. *Nat. Immunol.* **2008**, *9*, 166–175.

44. Graeber, K.E.; Olsen, N.J. Th17 cell cytokine secretion profile in host defense and autoimmunity. *Inflamm. Res. Off. J. Eur. Histamine Res. Soc. Al* **2012**, *61*, 87–96.

45. Ding, Y.; Li, J.; Wu, Q.; Yang, P.; Luo, B.; Xie, S.; Druey, K.M.; Zajac, A.J.; Hsu, H.-C.; Mountz, J.D. IL-17RA is essential for optimal localization of follicular Th cells in the germinal center light zone to promote autoantibody-producing B cells. *J. Immunol. Baltim. Md 1950* **2013**, *191*, 1614–1624.

46. Niu, X.; He, D.; Zhang, X.; Yue, T.; Li, N.; Zhang, J.Z.; Dong, C.; Chen, G. IL-21 regulates Th17 cells in rheumatoid arthritis. *Hum. Immunol.* **2010**, *71*, 334–341.

47. Schuurman, H.J.; Bell, E.B.; Gärtner, K.; Hedrich, H.J.; Hansen, A.K.; Kruijt, B.C. de Vrey, P.; Leyten, R.; Maeder, S.J.; Moutier, R. Comparative evaluation of the immune status of congenitally athymic and euthymic rat strains bred and maintained at different institutes: 2. Athymic rats. *J. Exp. Anim. Sci.* **1992**, *35*, 33–48.

48. Dianda, L.; Gulbranson-Judge, A.; Pao, W.; Hayday, A.C.; MacLennan, I.C.; Owen, M.J. Germinal center formation in mice lacking alpha beta T cells. *Eur. J. Immunol.* **1996**, *26*, 1603–1607.

49. Förster, R.; Schubel, A.; Breitfeld, D.; Kremmer, E.; Renner-Müller, I.; Wolf, E.; Lipp, M. CCR7 coordinates the primary immune response by establishing functional microenvironments in secondary lymphoid organs. *Cell* **1999**, *99*, 23–33.

50. Hardtke, S.; Ohl, L.; Förster, R. Balanced expression of CXCR5 and CCR7 on follicular T helper cells determines their transient positioning to lymph node follicles and is essential for efficient B-cell help. *Blood* **2005**, *106*, 1924–1931.

51. Haynes, N.M.; Allen, C.D.C.; Lesley, R.; Ansel, K.M.; Killeen, N.; Cyster, J.G. Role of CXCR5 and CCR7 in follicular Th cell positioning and appearance of a programmed cell death gene-1high germinal center-associated subpopulation. *J. Immunol. Baltim. Md 1950* **2007**, *179*, 5099–5108.

52. Förster, R.; Mattis, A.E.; Kremmer, E.; Wolf, E.; Brem, G.; Lipp, M. A putative chemokine receptor, BLR1, directs B cell migration to defined lymphoid organs and specific anatomic compartments of the spleen. *Cell* **1996**, *87*, 1037–1047.

53. Allen, C.D.C.; Ansel, K.M.; Low, C.; Lesley, R.; Tamamura, H.; Fujii, N.; Cyster, J.G. Germinal center dark and light zone organization is mediated by CXCR4 and CXCR5. *Nat. Immunol.* **2004**, *5*, 943–952.

54. Foy, T.M.; Laman, J.D.; Ledbetter, J.A.; Aruffo, A.; Claassen, E.; Noelle, R.J. gp39-CD40 interactions are essential for germinal center formation and the development of B cell memory. *J. Exp. Med.* **1994**, *180*, 157–163.

55. Renshaw, B.R.; Fanslow, W.C.; Armitage, R.J.; Campbell, K.A.; Liggitt, D.; Wright, B.; Davison, B.L.; Maliszewski, C.R. Humoral immune responses in CD40 ligand-deficient mice. *J. Exp. Med.* **1994**, *180*, 1889–1900.

56. Han, S.; Hathcock, K.; Zheng, B.; Kepler, T.B.; Hodes, R.; Kelsoe, G. Cellular interaction in germinal centers. Roles of CD40 ligand and B7-2 in established germinal centers. *J. Immunol. Baltim. Md 1950* **1995**, *155*, 556–567.

57. van Essen, D.; Kikutani, H.; Gray, D. CD40 ligand-transduced co-stimulation of T cells in the development of helper function. *Nature* **1995**, *378*, 620–623.

58. Kim, C.H.; Lim, H.W.; Kim, J.R.; Rott, L.; Hillsamer, P.; Butcher, E.C. Unique gene expression program of human germinal center T helper cells. *Blood* **2004**, *104*, 1952–1960.

59. Tafuri, A.; Shahinian, A.; Bladt, F.; Yoshinaga, S.K.; Jordana, M.; Wakeham, A.; Boucher, L.M.; Bouchard, D.; Chan, V.S.; Duncan, G.; *et al.* ICOS is essential for effective T-helper-cell responses. *Nature* **2001**, *409*, 105–109.

60. Ettinger, R.; Sims, G.P.; Fairhurst, A.-M.; Robbins, R.; da Silva, Y.S.; Spolski, R.; Leonard, W.J.; Lipsky, P.E. IL-21 induces differentiation of human naive and memory B cells into antibody-secreting plasma cells. *J. Immunol. Baltim. Md 1950* **2005**, *175*, 7867–7879.

61. Bryant, V.L.; Ma, C.S.; Avery, D.T.; Li, Y.; Good, K.L.; Corcoran, L.M. de Waal Malefyt, R.; Tangye, S.G. Cytokine-mediated regulation of human B cell differentiation into Ig-secreting cells: Predominant role of IL-21 produced by CXCR5+ T follicular helper cells. *J. Immunol. Baltim. Md 1950* **2007**, *179*, 8180–8190.

62. Vinuesa, C.G.; Cook, M.C.; Angelucci, C.; Athanasopoulos, V.; Rui, L.; Hill, K.M.; Yu, D.; Domaschenz, H.; Whittle, B.; Lambe, T.; *et al*. A RING-type ubiquitin ligase family member required to repress follicular helper T cells and autoimmunity. *Nature* **2005**, *435*, 452–458.

63. Rasheed, A.-U.; Rahn, H.-P.; Sallusto, F.; Lipp, M.; Müller, G. Follicular B helper T cell activity is confined to CXCR5(hi)ICOS(hi) CD4 T cells and is independent of CD57 expression. *Eur. J. Immunol.* **2006**, *36*, 1892–1903.

64. Morita, R.; Schmitt, N.; Bentebibel, S.-E.; Ranganathan, R.; Bourdery, L.; Zurawski, G.; Foucat, E.; Dullaers, M.; Oh, S.; Sabzghabaei, N.; *et al*. Human blood CXCR5(+)CD4(+) T cells are counterparts of T follicular cells and contain specific subsets that differentially support antibody secretion. *Immunity* **2011**, *34*, 108–121.

65. Sage, P.T.; Francisco, L.M.; Carman, C.V.; Sharpe, A.H. The receptor PD-1 controls follicular regulatory T cells in the lymph nodes and blood. *Nat. Immunol.* **2013**, *14*, 152–161.

66. MacLeod, M.K.L.; David, A.; McKee, A.S.; Crawford, F.; Kappler, J.W.; Marrack, P. Memory CD4 T cells that express CXCR5 provide accelerated help to B cells. *J. Immunol. Baltim. Md 1950* **2011**, *186*, 2889–2896.

67. Weber, J.P.; Fuhrmann, F.; Hutloff, A. T-follicular helper cells survive as long-term memory cells. *Eur. J. Immunol.* **2012**, *42*, 1981–1988.

68. Hale, J.S.; Youngblood, B.; Latner, D.R.; Mohammed, A.U.R.; Ye, L.; Akondy, R.S.; Wu, T.; Iyer, S.S.; Ahmed, R. Distinct memory CD4+ T cells with commitment to T follicular helper- and T helper 1-cell lineages are generated after acute viral infection. *Immunity* **2013**, *38*, 805–817.

69. Nurieva, R.I.; Chung, Y.; Martinez, G.J.; Yang, X.O.; Tanaka, S.; Matskevitch, T.D.; Wang, Y.-H.; Dong, C. Bcl6 mediates the development of T follicular helper cells. *Science* **2009**, *325*, 1001–1005.

70. Yu, D.; Rao, S.; Tsai, L.M.; Lee, S.K.; He, Y.; Sutcliffe, E.L.; Srivastava, M.; Linterman, M.; Zheng, L.; Simpson, N.; *et al*. The transcriptional repressor Bcl-6 directs T follicular helper cell lineage commitment. *Immunity* **2009**, *31*, 457–468.

71. Fazilleau, N.; McHeyzer-Williams, L.J.; Rosen, H.; McHeyzer-Williams, M.G. The function of follicular helper T cells is regulated by the strength of T cell antigen receptor binding. *Nat. Immunol.* **2009**, *10*, 375–384.

72. Junt, T.; Fink, K.; Förster, R.; Senn, B.; Lipp, M.; Muramatsu, M.; Zinkernagel, R.M.; Ludewig, B.; Hengartner, H. CXCR5-dependent seeding of follicular niches by B and Th cells augments antiviral B cell responses. *J. Immunol. Baltim. Md 1950* **2005**, *175*, 7109–7116.

73. Poholek, A.C.; Hansen, K.; Hernandez, S.G.; Eto, D.; Chandele, A.; Weinstein, J.S.; Dong, X.; Odegard, J.M.; Kaech, S.M.; Dent, A.L.; *et al*. *In vivo* regulation of Bcl6 and T follicular helper cell development. *J. Immunol. Baltim. Md 1950* **2010**, *185*, 313–326.

74. Choi, Y.S.; Kageyama, R.; Eto, D.; Escobar, T.C.; Johnston, R.J.; Monticelli, L.; Lao, C.; Crotty, S. ICOS receptor instructs T follicular helper cell *versus* effector cell differentiation via induction of the transcriptional repressor Bcl6. *Immunity* **2011**, *34*, 932–946.

75. Kerfoot, S.M.; Yaari, G.; Patel, J.R.; Johnson, K.L.; Gonzalez, D.G.; Kleinstein, S.H.; Haberman, A.M. Germinal center B cell and T follicular helper cell development initiates in the interfollicular zone. *Immunity* **2011**, *34*, 947–960.

76. Kitano, M.; Moriyama, S.; Ando, Y.; Hikida, M.; Mori, Y.; Kurosaki, T.; Okada, T. Bcl6 protein expression shapes pre-germinal center B cell dynamics and follicular helper T cell heterogeneity. *Immunity* **2011**, *34*, 961–972.

77. Celli, S.; Lemaître, F.; Bousso, P. Real-time manipulation of T cell-dendritic cell interactions *in vivo* reveals the importance of prolonged contacts for CD4+ T cell activation. *Immunity* **2007**, *27*, 625–634.

78. King, C.; Tangye, S.G.; Mackay, C.R. T follicular helper (TFH) cells in normal and dysregulated immune responses. *Annu. Rev. Immunol.* **2008**, *26*, 741–766.

79. Deenick, E.K.; Chan, A.; Ma, C.S.; Gatto, D.; Schwartzberg, P.L.; Brink, R.; Tangye, S.G. Follicular helper T cell differentiation requires continuous antigen presentation that is independent of unique B cell signaling. *Immunity* **2010**, *33*, 241–253.

80. Ron, Y.; Sprent, J. T cell priming *in vivo*: A major role for B cells in presenting antigen to T cells in lymph nodes. *J. Immunol. Baltim. Md 1950* **1987**, *138*, 2848–2856.

81. Baumjohann, D.; Okada, T.; Ansel, K.M. Cutting Edge: Distinct waves of BCL6 expression during T follicular helper cell development. *J. Immunol. Baltim. Md 1950* **2011**, *187*, 2089–2092.

82. Cannons, J.L.; Qi, H.; Lu, K.T.; Dutta, M.; Gomez-Rodriguez, J.; Cheng, J.; Wakeland, E.K.; Germain, R.N.; Schwartzberg, P.L. Optimal germinal center responses require a multistage T cell:B cell adhesion process involving integrins, SLAM-associated protein, and CD84. *Immunity* **2010**, *32*, 253–265.

83. Qi, H.; Cannons, J.L.; Klauschen, F.; Schwartzberg, P.L.; Germain, R.N. SAP-controlled T-B cell interactions underlie germinal centre formation. *Nature* **2008**, *455*, 764–769.

84. Xu, H.; Li, X.; Liu, D.; Li, J.; Zhang, X.; Chen, X.; Hou, S.; Peng, L.; Xu, C.; Liu, W.; Zhang, L.; Qi, H. Follicular T-helper cell recruitment governed by bystander B cells and ICOS-driven motility. *Nature* **2013**, *496*, 523–527.

85. Johnston, R.J.; Poholek, A.C.; DiToro, D.; Yusuf, I.; Eto, D.; Barnett, B.; Dent, A.L.; Craft, J.; Crotty, S. Bcl6 and Blimp-1 are reciprocal and antagonistic regulators of T follicular helper cell differentiation. *Science* **2009**, *325*, 1006–1010.

86. Choi, Y.S.; Eto, D.; Yang, J.A.; Lao, C.; Crotty, S. Cutting edge: STAT1 is required for IL-6-mediated Bcl6 induction for early follicular helper cell differentiation. *J. Immunol. Baltim. Md 1950* **2013**, *190*, 3049–3053.

87. Kroenke, M.A.; Eto, D.; Locci, M.; Cho, M.; Davidson, T.; Haddad, E.K.; Crotty, S. Bcl6 and Maf cooperate to instruct human follicular helper CD4 T cell differentiation. *J. Immunol. Baltim. Md 1950* **2012**, *188*, 3734–3744.

88. Klein, U.; Dalla-Favera, R. Germinal centres: Role in B-cell physiology and malignancy. *Nat. Rev. Immunol.* **2008**, *8*, 22–33.

89. Martins, G.; Calame, K. Regulation and functions of Blimp-1 in T and B lymphocytes. *Annu. Rev. Immunol.* **2008**, *26*, 133–169.

90. Bauquet, A.T.; Jin, H.; Paterson, A.M.; Mitsdoerffer, M.; Ho, I.-C.; Sharpe, A.H.; Kuchroo, V.K. The costimulatory molecule ICOS regulates the expression of c-Maf and IL-21 in the development of follicular T helper cells and TH-17 cells. *Nat. Immunol.* **2009**, *10*, 167–175.

91. Hiramatsu, Y.; Suto, A.; Kashiwakuma, D.; Kanari, H.; Kagami, S.; Ikeda, K.; Hirose, K.; Watanabe, N.; Grusby, M.J.; Iwamoto, I.; *et al*. c-Maf activates the promoter and enhancer of the IL-21 gene, and TGF-beta inhibits c-Maf-induced IL-21 production in CD4+ T cells. *J. Leukoc. Biol.* **2010**, *87*, 703–712.

92. Kwon, H.; Thierry-Mieg, D.; Thierry-Mieg, J.; Kim, H.-P.; Oh, J.; Tunyaplin, C.; Carotta, S.; Donovan, C.E.; Goldman, M.L.; Tailor, P.; *et al*. Analysis of interleukin-21-induced Prdm1 gene regulation reveals functional cooperation of STAT3 and IRF4 transcription factors. *Immunity* **2009**, *31*, 941–952.

93. Bollig, N.; Brüstle, A.; Kellner, K.; Ackermann, W.; Abass, E.; Raifer, H.; Camara, B.; Brendel, C.; Giel, G.; Bothur, E.; *et al*. Transcription factor IRF4 determines germinal center formation through follicular T-helper cell differentiation. *Proc. Natl. Acad. Sci. USA* **2012**, *109*, 8664–8669.

94. Lohoff, M.; Mittrücker, H.-W.; Prechtl, S.; Bischof, S.; Sommer, F.; Kock, S.; Ferrick, D.A.; Duncan, G.S.; Gessner, A.; Mak, T.W. Dysregulated T helper cell differentiation in the absence of interferon regulatory factor 4. *Proc. Natl. Acad. Sci. USA* **2002**, *99*, 11808–11812.

95. Brüstle, A.; Heink, S.; Huber, M.; Rosenplänter, C.; Stadelmann, C.; Yu, P.; Arpaia, E.; Mak, T.W.; Kamradt, T.; Lohoff, M. The development of inflammatory T(H)-17 cells requires interferon-regulatory factor 4. *Nat. Immunol.* **2007**, *8*, 958–966.

96. Staudt, V.; Bothur, E.; Klein, M.; Lingnau, K.; Reuter, S.; Grebe, N.; Gerlitzki, B.; Hoffmann, M.; Ulges, A.; Taube, C.; *et al*. Interferon-regulatory factor 4 is essential for the developmental program of T helper 9 cells. *Immunity* **2010**, *33*, 192–202.

97. Serre, K.; Mohr, E.; Bénézech, C.; Bird, R.; Khan, M.; Caamaño, J.H.; Cunningham, A.F.; Maclennan, I.C.M. Selective effects of NF-κB1 deficiency in CD4^+^ T cells on Th2 and TFh induction by alum-precipitated protein vaccines. *Eur. J. Immunol.* **2011**, *41*, 1573–1582.

98. Auderset, F.; Schuster, S.; Fasnacht, N.; Coutaz, M.; Charmoy, M.; Koch, U.; Favre, S.; Wilson, A.; Trottein, F.; Alexander, J.; *et al*. Notch signaling regulates follicular helper T cell differentiation. *J. Immunol. Baltim. Md 1950* **2013**, *191*, 2344–2350.

99. Liu, X.; Chen, X.; Zhong, B.; Wang, A.; Wang, X.; Chu, F.; Nurieva, R.I.; Yan, X.; Chen, P.; van der Flier, L.G.; *et al*. Transcription factor achaete-scute homologue 2 initiates follicular T-helper-cell development. *Nature* **2014**, *507*, 513–518.

100. Shevach, E.M. Regulatory T Cells in Autoimmmunity. *Annu. Rev. Immunol.* **2000**, *18*, 423–449.

101. Lim, H.W.; Hillsamer, P.; Banham, A.H.; Kim, C.H. Cutting edge: Direct suppression of B cells by CD4+ CD25+ regulatory T cells. *J. Immunol. Baltim. Md 1950* **2005**, *175*, 4180–4183.

102. Lim, H.W.; Hillsamer, P.; Kim, C.H. Regulatory T cells can migrate to follicles upon T cell activation and suppress GC-Th cells and GC-Th cell-driven B cell responses. *J. Clin. Investig.* **2004**, *114*, 1640–1649.

103. Chung, Y.; Tanaka, S.; Chu, F.; Nurieva, R.I.; Martinez, G.J.; Rawal, S.; Wang, Y.-H.; Lim, H.; Reynolds, J.M.; Zhou, X.; *et al*. Follicular regulatory T cells expressing Foxp3 and Bcl-6 suppress germinal center reactions. *Nat. Med.* **2011**, *17*, 983–988.

104. Wollenberg, I.; Agua-Doce, A.; Hernández, A.; Almeida, C.; Oliveira, V.G.; Faro, J.; Graca, L. Regulation of the germinal center reaction by Foxp3+ follicular regulatory T cells. *J. Immunol. Baltim. Md 1950* **2011**, *187*, 4553–4560.

105. Barrat, F.J.; Cua, D.J.; Boonstra, A.; Richards, D.F.; Crain, C.; Savelkoul, H.F.; de Waal-Malefyt, R.; Coffman, R.L.; Hawrylowicz, C.M.; O’Garra, A. *In vitro* generation of interleukin 10-producing regulatory CD4(+) T cells is induced by immunosuppressive drugs and inhibited by T helper type 1 (Th1)- and Th2-inducing cytokines. *J. Exp. Med.* **2002**, *195*, 603–616.

106. Horwitz, D.A.; Zheng, S.G.; Gray, J.D. Natural and TGF-β–induced Foxp3+CD4+ CD25+ regulatory T cells are not mirror images of each other. *Trends Immunol.* **2008**, *29*, 429–435.

107. Alexander, C.-M.; Tygrett, L.T.; Boyden, A.W.; Wolniak, K.L.; Legge, K.L.; Waldschmidt, T.J. T regulatory cells participate in the control of germinal centre reactions. *Immunology* **2011**, *133*, 452–468.

108. Josefowicz, S.Z.; Niec, R.E.; Kim, H.Y.; Treuting, P.; Chinen, T.; Zheng, Y.; Umetsu, D.T.; Rudensky, A.Y. Extrathymically generated regulatory T cells control mucosal TH2 inflammation. *Nature* **2012**, *482*, 395–399.

109. Sage, P.T.; Sharpe, A.H. T follicular regulatory cells in the regulation of B cell responses. *Trends Immunol.* **2015**, *36*, 410–418.

110. Ballesteros-Tato, A.; León, B.; Graf, B.A.; Moquin, A.; Adams, P.S.; Lund, F.E.; Randall, T.D. Interleukin-2 inhibits germinal center formation by limiting T follicular helper cell differentiation. *Immunity* **2012**, *36*, 847–856.

111. Peuchmaur, M.; Emilie, D.; Crevon, M.C.; Brousse, N.; Gaulard, P.; D’Agay, M.F.; Galanaud, P.; Solal-Celigny, P. Interleukin-2 and interferon-gamma production in follicular lymphomas. *Am. J. Clin. Pathol.* **1991**, *95*, 55–62.

112. Vyth-Dreese, F.A.; Boot, H.; Dellemijn, T.A.; Majoor, D.M.; Oomen, L.C.; Laman, J.D.; Van Meurs, M.; De Weger, R.A.; De Jong, D. Localization *in situ* of costimulatory molecules and cytokines in B-cell non-Hodgkin’s lymphoma. *Immunology* **1998**, *94*, 580–586.

113. Emilie, D.; Peuchmaur, M.; Maillot, M.C.; Crevon, M.C.; Brousse, N.; Delfraissy, J.F.; Dormont, J.; Galanaud, P. Production of interleukins in human immunodeficiency virus-1-replicating lymph nodes. *J. Clin. Investig.* **1990**, *86*, 148–159.

114. Heinen, E. *In Vivo Immunology : Regulatory Processes during Lymphopoiesis and Immunopoiesis*, Proceedings of the International Conference on Lymphatic Tissues and Germinal Centers in Immune Reactions 1993: Liege, B.; In Series Advances in Experimental Medicine and Biology; Springer: New York, NY, USA, 1994; Volume 355. 

115. Bogen, S.A.; Fogelman, I.; Abbas, A.K. Analysis of IL-2, IL-4, and IFN-gamma-producing cells *in situ* during immune responses to protein antigens. *J. Immunol. Baltim. Md 1950* **1993**, *150*, 4197–4205.

116. Butch, A.W.; Chung, G.H.; Hoffmann, J.W.; Nahm, M.H. Cytokine expression by germinal center cells. *J. Immunol. Baltim. Md 1950* **1993**, *150*, 39–47.

117. Toellner, K.M.; Scheel-Toellner, D.; Sprenger, R.; Duchrow, M.; Trümper, L.H.; Ernst, M.; Flad, H.D.; Gerdes, J. The human germinal centre cells, follicular dendritic cells and germinal centre T cells produce B cell-stimulating cytokines. *Cytokine* **1995**, *7*, 344–354.

118. King, I.L.; Mohrs, M. IL-4-producing CD4+ T cells in reactive lymph nodes during helminth infection are T follicular helper cells. *J. Exp. Med.* **2009**, *206*, 1001–1007.

119. Zaretsky, A.G.; Taylor, J.J.; King, I.L.; Marshall, F.A.; Mohrs, M.; Pearce, E.J. T follicular helper cells differentiate from Th2 cells in response to helminth antigens. *J. Exp. Med.* **2009**, *206*, 991–999.

120. Fazilleau, N.; Eisenbraun, M.D.; Malherbe, L.; Ebright, J.N.; Pogue-Caley, R.R.; McHeyzer-Williams, L.J.; McHeyzer-Williams, M.G. Lymphoid reservoirs of antigen-specific memory T helper cells. *Nat. Immunol.* **2007**, *8*, 753–761.

121. Lüthje, K.; Kallies, A.; Shimohakamada, Y.; Belz, G.T.; Light, A.; Tarlinton, D.M.; Nutt, S.L. The development and fate of follicular helper T cells defined by an IL-21 reporter mouse. *Nat. Immunol.* **2012**, *13*, 491–498.

122. Tsuji, M.; Komatsu, N.; Kawamoto, S.; Suzuki, K.; Kanagawa, O.; Honjo, T.; Hori, S.; Fagarasan, S. Preferential generation of follicular B helper T cells from Foxp3+ T cells in gut Peyer’s patches. *Science* **2009**, *323*, 1488–1492.

123. Kopf, M.; Herren, S.; Wiles, M.V.; Pepys, M.B.; Kosco-Vilbois, M.H. Interleukin 6 influences germinal center development and antibody production via a contribution of C3 complement component. *J. Exp. Med.* **1998**, *188*, 1895–1906.

124. McGuire, H.M.; Vogelzang, A.; Warren, J.; Loetsch, C.; Natividad, K.D.; Chan, T.D.; Brink, R.; Batten, M.; King, C. IL-21 and IL-4 Collaborate To Shape T-Dependent Antibody Responses. *J. Immunol. Baltim. Md*. *1950.* **2015**, *195*, 5123-5135.

125. Chavele, K.-M.; Merry, E.; Ehrenstein, M.R. Cutting edge: Circulating plasmablasts induce the differentiation of human T follicular helper cells via IL-6 production. *J. Immunol. Baltim. Md 1950* **2015**, *194*, 2482–2485.

126. Wu, Y.; El Shikh, M.E.M.; El Sayed, R.M.; Best, A.M.; Szakal, A.K.; Tew, J.G. IL-6 produced by immune complex-activated follicular dendritic cells promotes germinal center reactions, IgG responses and somatic hypermutation. *Int. Immunol.* **2009**, *21*, 745–756.

127. Ozaki, K.; Kikly, K.; Michalovich, D.; Young, P.R.; Leonard, W.J. Cloning of a type I cytokine receptor most related to the IL-2 receptor beta chain. *Proc. Natl. Acad. Sci. USA* **2000**, *97*, 11439–11444.

128. Kasaian, M.T.; Whitters, M.J.; Carter, L.L.; Lowe, L.D.; Jussif, J.M.; Deng, B.; Johnson, K.A.; Witek, J.S.; Senices, M.; Konz, R.F.; *et al*. IL-21 limits NK cell responses and promotes antigen-specific T cell activation: A mediator of the transition from innate to adaptive immunity. *Immunity* **2002**, *16*, 559–569.

129. Avery, D.T.; Deenick, E.K.; Ma, C.S.; Suryani, S.; Simpson, N.; Chew, G.Y.; Chan, T.D.; Palendira, U.; Bustamante, J.; Boisson-Dupuis, S.; *et al*. B cell–intrinsic signaling through IL-21 receptor and STAT3 is required for establishing long-lived antibody responses in humans. *J. Exp. Med.* **2010**, *207*, 155 –171.

130. Zeng, R.; Spolski, R.; Casas, E.; Zhu, W.; Levy, D.E.; Leonard, W.J. The molecular basis of IL-21-mediated proliferation. *Blood* **2007**, *109*, 4135–4142.

131. Asao, H.; Okuyama, C.; Kumaki, S.; Ishii, N.; Tsuchiya, S.; Foster, D.; Sugamura, K. Cutting edge: The common gamma-chain is an indispensable subunit of the IL-21 receptor complex. *J. Immunol. Baltim. Md 1950* **2001**, *167*, 1–5.

132. Delgoffe, G.M.; Vignali, D.A.A. STAT heterodimers in immunity: A mixed message or a unique signal? *JAK-STAT* **2013**, *2*, e23060.

133. Heinrich, P.C.; Behrmann, I.; Müller-Newen, G.; Schaper, F.; Graeve, L. Interleukin-6-type cytokine signalling through the gp130/Jak/STAT pathway. *Biochem. J.* **1998**, *334( Pt 2)*, 297–314.

134. Eddahri, F.; Denanglaire, S.; Bureau, F.; Spolski, R.; Leonard, W.J.; Leo, O.; Andris, F. Interleukin-6/STAT3 signaling regulates the ability of naive T cells to acquire B-cell help capacities. *Blood* **2009**, *113*, 2426–2433.

135. Fornek, J.L.; Tygrett, L.T.; Waldschmidt, T.J.; Poli, V.; Rickert, R.C.; Kansas, G.S. Critical role for Stat3 in T-dependent terminal differentiation of IgG B cells. *Blood* **2006**, *107*, 1085–1091.

136. Ozaki, K.; Spolski, R.; Ettinger, R.; Kim, H.-P.; Wang, G.; Qi, C.-F.; Hwu, P.; Shaffer, D.J.; Akilesh, S.; Roopenian, D.C.; *et al*. Regulation of B cell differentiation and plasma cell generation by IL-21, a novel inducer of Blimp-1 and Bcl-6. *J. Immunol. Baltim. Md 1950* **2004**, *173*, 5361–5371.

137. Lee, S.K.; Rigby, R.J.; Zotos, D.; Tsai, L.M.; Kawamoto, S.; Marshall, J.L.; Ramiscal, R.R.; Chan, T.D.; Gatto, D.; Brink, R.; *et al*. B cell priming for extrafollicular antibody responses requires Bcl-6 expression by T cells. *J. Exp. Med.* **2011**, *208*, 1377–1388.

138. Dienz, O.; Eaton, S.M.; Bond, J.P.; Neveu, W.; Moquin, D.; Noubade, R.; Briso, E.M.; Charland, C.; Leonard, W.J.; Ciliberto, G.; Teuscher, C.; Haynes, L.; Rincon, M. The induction of antibody production by IL-6 is indirectly mediated by IL-21 produced by CD4+ T cells. *J. Exp. Med.* **2009**, *206*, 69–78.

139. Batten, M.; Ghilardi, N. The biology and therapeutic potential of interleukin 27. *J. Mol. Med.* **2007**, *85*, 661–672.

140. Shields, D.C.; Harmon, D.L.; Nunez, F.; Whitehead, A.S. The evolution of haematopoietic cytokine/receptor complexes. *Cytokine* **1995**, *7*, 679–688.

141. Fontenot, J.D.; Rasmussen, J.P.; Gavin, M.A.; Rudensky, A.Y. A function for interleukin 2 in Foxp3-expressing regulatory T cells. *Nat. Immunol.* **2005**, *6*, 1142–1151.

142. Willerford, D.M.; Chen, J.; Ferry, J.A.; Davidson, L.; Ma, A.; Alt, F.W. Interleukin-2 receptor alpha chain regulates the size and content of the peripheral lymphoid compartment. *Immunity* **1995**, *3*, 521–530.

143. Attridge, K.; Wang, C.J.; Wardzinski, L.; Kenefeck, R.; Chamberlain, J.L.; Manzotti, C.; Kopf, M.; Walker, L.S. K. IL-21 inhibits T cell IL-2 production and impairs Treg homeostasis. *Blood* **2012**, *119*, 4656–4664.

144. Schmitz, I.; Schneider, C.; Fröhlich, A.; Frebel, H.; Christ, D.; Leonard, W.J.; Sparwasser, T.; Oxenius, A.; Freigang, S.; Kopf, M. IL-21 restricts virus-driven Treg cell expansion in chronic LCMV infection. *PLoS Pathog.* **2013**, *9*, e1003362.

145. Bennett, C.L.; Ochs, H.D. IPEX is a unique X-linked syndrome characterized by immune dysfunction, polyendocrinopathy, enteropathy, and a variety of autoimmune phenomena. *Curr. Opin. Pediatr.* **2001**, *13*, 533–538.

146. Ramsdell, F.; Ziegler, S.F. FOXP3 and scurfy: How it all began. *Nat. Rev. Immunol.* **2014**, *14*, 343–349.

147. Nurieva, R.; Yang, X.O.; Martinez, G.; Zhang, Y.; Panopoulos, A.D.; Ma, L.; Schluns, K.; Tian, Q.; Watowich, S.S.; Jetten, A.M.; *et al*. Essential autocrine regulation by IL-21 in the generation of inflammatory T cells. *Nature* **2007**, *448*, 480–483.

148. Korn, T.; Bettelli, E.; Gao, W.; Awasthi, A.; Jäger, A.; Strom, T.B.; Oukka, M.; Kuchroo, V.K. IL-21 initiates an alternative pathway to induce proinflammatory T(H)17 cells. *Nature* **2007**, *448*, 484–487.

149. Vogelzang, A.; McGuire, H.M.; Liu, S.M.; Gloss, B.; Mercado, K.; Earls, P.; Dinger, M.E.; Batten, M.; Sprent, J.; King, C. IL-21 contributes to fatal inflammatory disease in the absence of Foxp3+ T regulatory cells. *J. Immunol. Baltim. Md 1950* **2014**, *192*, 1404–1414.

150. Clough, L.E.; Wang, C.J.; Schmidt, E.M.; Booth, G.; Hou, T.Z.; Ryan, G.A.; Walker, L.S.K. Release from regulatory T cell-mediated suppression during the onset of tissue-specific autoimmunity is associated with elevated IL-21. *J. Immunol. Baltim. Md 1950* **2008**, *180*, 5393–5401.

151. Li, Y.; Yee, C. IL-21 mediated Foxp3 suppression leads to enhanced generation of antigen-specific CD8+ cytotoxic T lymphocytes. *Blood* **2008**, *111*, 229–235.

152. Piao, W.-H.; Jee, Y.H.; Liu, R.L.; Coons, S.W.; Kala, M.; Collins, M.; Young, D.A.; Campagnolo, D.I.; Vollmer, T.L.; Bai, X.-F.; *et al*. IL-21 modulates CD4+ CD25+ regulatory T-cell homeostasis in experimental autoimmune encephalomyelitis. *Scand. J. Immunol.* **2008**, *67*, 37–46.

153. Johnston, R.J.; Choi, Y.S.; Diamond, J.A.; Yang, J.A.; Crotty, S. STAT5 is a potent negative regulator of TFH cell differentiation. *J. Exp. Med.* **2012**, *209*, 243–250.

154. Nurieva, R.I.; Podd, A.; Chen, Y.; Alekseev, A.M.; Yu, M.; Qi, X.; Huang, H.; Wen, R.; Wang, J.; Li, H.S.; *et al*. STAT5 protein negatively regulates T follicular helper (Tfh) cell generation and function. *J. Biol. Chem.* **2012**, *287*, 11234–11239.

155. Ding, Y.; Li, J.; Yang, P.; Luo, B.; Wu, Q.; Zajac, A.J.; Wildner, O.; Hsu, H.-C.; Mountz, J.D. Interleukin-21 Promotes Germinal Center Reaction by Skewing the Follicular Regulatory T Cell to Follicular Helper T Cell Balance in Autoimmune BXD2 Mice. *Arthritis Rheumatol. Hoboken NJ* **2014**, *66*, 2601–2612.

156. Sahoo, A.; Alekseev, A.; Tanaka, K.; Obertas, L.; Lerman, B.; Haymaker, C.; Clise-Dwyer, K.; McMurray, J.S.; Nurieva, R. Batf is important for IL-4 expression in T follicular helper cells. *Nat. Commun.* **2015**, *6*:7997.

157. Betz, B.C.; Jordan-Williams, K.L.; Wang, C.; Kang, S.G.; Liao, J.; Logan, M.R.; Kim, C.H.; Taparowsky, E.J. Batf coordinates multiple aspects of B and T cell function required for normal antibody responses. *J. Exp. Med.* **2010**, *207*, 933–942.

158. Ise, W.; Kohyama, M.; Schraml, B.U.; Zhang, T.; Schwer, B.; Basu, U.; Alt, F.W.; Tang, J.; Oltz, E.M.; Murphy, T.L.; Murphy, K.M. Batf controls the global regulators of class switch recombination in both B and T cells. *Nat. Immunol.* **2011**, *12*, 536–543.

159. Pène, J.; Gauchat, J.-F.; Lécart, S.; Drouet, E.; Guglielmi, P.; Boulay, V.; Delwail, A.; Foster, D.; Lecron, J.-C.; Yssel, H. Cutting edge: IL-21 is a switch factor for the production of IgG1 and IgG3 by human B cells. *J. Immunol. Baltim. Md 1950* **2004**, *172*, 5154–5157.

160. Pène, J.; Guglielmi, L.; Gauchat, J.-F.; Harrer, N.; Woisetschläger, M.; Boulay, V.; Fabre, J.-M.; Demoly, P.; Yssel, H. IFN-gamma-mediated inhibition of human IgE synthesis by IL-21 is associated with a polymorphism in the IL-21R gene. *J. Immunol. Baltim. Md 1950* **2006**, *177*, 5006–5013.

161. Kotlarz, D.; Ziętara, N.; Uzel, G.; Weidemann, T.; Braun, C.J.; Diestelhorst, J.; Krawitz, P.M.; Robinson, P.N.; Hecht, J.; Puchałka, J.; *et al*. Loss-of-function mutations in the IL-21 receptor gene cause a primary immunodeficiency syndrome. *J. Exp. Med.* **2013**, *210*, 433–443.

162. Suto, A.; Nakajima, H.; Hirose, K.; Suzuki, K.; Kagami, S.; Seto, Y.; Hoshimoto, A.; Saito, Y.; Foster, D.C.; Iwamoto, I. Interleukin 21 prevents antigen-induced IgE production by inhibiting germ line C(epsilon) transcription of IL-4-stimulated B cells. *Blood* **2002**, *100*, 4565–4573.

163. Kitayama, D.; Sakamoto, A.; Arima, M.; Hatano, M.; Miyazaki, M.; Tokuhisa, T. A role for Bcl6 in sequential class switch recombination to IgE in B cells stimulated with IL-4 and IL-21. *Mol. Immunol.* **2008**, *45*, 1337–1345.

164. Ma, C.S.; Suryani, S.; Avery, D.T.; Chan, A.; Nanan, R.; Santner-Nanan, B.; Deenick, E.K.; Tangye, S.G. Early commitment of naïve human CD4(+) T cells to the T follicular helper (T(FH)) cell lineage is induced by IL-12. *Immunol. Cell Biol.* **2009**, *87*, 590–600.

165. Yusuf, I.; Kageyama, R.; Monticelli, L.; Johnston, R.J.; Ditoro, D.; Hansen, K.; Barnett, B.; Crotty, S. Germinal center T follicular helper cell IL-4 production is dependent on signaling lymphocytic activation molecule receptor (CD150). *J. Immunol. Baltim. Md 1950* **2010**, *185*, 190–202.

166. Lee, S.K.; Silva, D.G.; Martin, J.L.; Pratama, A.; Hu, X.; Chang, P.-P.; Walters, G.; Vinuesa, C.G. Interferon-γ Excess Leads to Pathogenic Accumulation of Follicular Helper T Cells and Germinal Centers. *Immunity* **2012**, *37*, 880–892.

167. Conti, P.; Kempuraj, D.; Kandere, K.; Di Gioacchino, M.; Barbacane, R.C.; Castellani, M.L.; Felaco, M.; Boucher, W.; Letourneau, R.; Theoharides, T.C. IL-10, an inflammatory/inhibitory cytokine, but not always. *Immunol. Lett.* **2003**, *86*, 123–129.

168. Ebert, L.M.; Horn, M.P.; Lang, A.B.; Moser, B. B cells alter the phenotype and function of follicular-homing CXCR5+ T cells. *Eur. J. Immunol.* **2004**, *34*, 3562–3571.

169. Löhning, M.; Hutloff, A.; Kallinich, T.; Mages, H.W.; Bonhagen, K.; Radbruch, A.; Hamelmann, E.; Kroczek, R.A. Expression of ICOS *in vivo* defines CD4+ effector T cells with high inflammatory potential and a strong bias for secretion of interleukin 10. *J. Exp. Med.* **2003**, *197*, 181–193.

170. Arpin, C.; Déchanet, J.; Van Kooten, C.; Merville, P.; Grouard, G.; Brière, F.; Banchereau, J.; Liu, Y.J. Generation of memory B cells and plasma cells *in vitro*. *Science* **1995**, *268*, 720–722.

171. Balasa, B.; Van Gunst, K.; Jung, N.; Balakrishna, D.; Santamaria, P.; Hanafusa, T.; Itoh, N.; Sarvetnick, N. Islet-specific expression of IL-10 promotes diabetes in nonobese diabetic mice independent of Fas, perforin, TNF receptor-1, and TNF receptor-2 molecules. *J. Immunol. Baltim. Md 1950* **2000**, *165*, 2841–2849.

172. Cai, G.; Nie, X.; Zhang, W.; Wu, B.; Lin, J.; Wang, H.; Jiang, C.; Shen, Q. A Regulatory Role for IL-10 Receptor Signaling in Development and B Cell Help of T Follicular Helper Cells in Mice. *J. Immunol.* **2012**, *189*, 1294–1302.

173. Wu, H.Y.; Quintana, F.J.; Weiner, H.L. Nasal anti-CD3 antibody ameliorates lupus by inducing an IL-10-secreting CD4+ CD25- LAP+ regulatory T cell and is associated with down-regulation of IL-17+ CD4+ ICOS+ CXCR5+ follicular helper T cells. *J. Immunol. Baltim. Md 1950* **2008**, *181*, 6038–6050.

174. Tangye, S.G.; Ma, C.S.; Brink, R.; Deenick, E.K. The good, the bad and the ugly - TFH cells in human health and disease. *Nat. Rev. Immunol.* **2013**, *13*, 412–426.

175. Moudgil, K.D.; Choubey, D. Cytokines in autoimmunity: Role in induction, regulation, and treatment. *J. Interferon Cytokine Res. Off. J. Int. Soc. Interferon Cytokine Res.* **2011**, *31*, 695–703.

176. Sweet, R.A.; Lee, S.K.; Vinuesa, C.G. Developing connections amongst key cytokines and dysregulated germinal centers in autoimmunity. *Curr. Opin. Immunol.* **2012**, *24*, 658–664.

177. Shi, G.-X.; Harrison, K.; Wilson, G.L.; Moratz, C.; Kehrl, J.H. RGS13 regulates germinal center B lymphocytes responsiveness to CXC chemokine ligand (CXCL)12 and CXCL13. *J. Immunol. Baltim. Md 1950* **2002**, *169*, 2507–2515.

178. Han, J.-I.; Huang, N.-N.; Kim, D.-U.; Kehrl, J.H. RGS1 and RGS13 mRNA silencing in a human B lymphoma line enhances responsiveness to chemoattractants and impairs desensitization. *J. Leukoc. Biol.* **2006**, *79*, 1357–1368.

179. Korn, T.; Bettelli, E.; Oukka, M.; Kuchroo, V.K. IL-17 and Th17 Cells. *Annu. Rev. Immunol.* **2009**, *27*, 485–517.

180. Mitsdoerffer, M.; Lee, Y.; Jäger, A.; Kim, H.-J.; Korn, T.; Kolls, J.K.; Cantor, H.; Bettelli, E.; Kuchroo, V.K. Proinflammatory T helper type 17 cells are effective B-cell helpers. *Proc. Natl. Acad. Sci. USA* **2010**, *107*, 14292–14297.

181. Hirota, K.; Turner, J.-E.; Villa, M.; Duarte, J.H.; Demengeot, J.; Steinmetz, O.M.; Stockinger, B. Plasticity of Th17 cells in Peyer’s patches is responsible for the induction of T cell-dependent IgA responses. *Nat. Immunol.* **2013**, *14*, 372–379.

182. Wichner, K.; Stauss, D.; Kampfrath, B.; Krüger, K.; Müller, G.; Rehm, A.; Lipp, M.; Höpken, U.E. Dysregulated development of IL-17-and IL-21-expressing follicular helper T cells and increased germinal center formation in the absence of RORγt. *FASEB J. Off. Publ. Fed. Am. Soc. Exp. Biol.* **2015**.

183. Noguchi, M.; Yi, H.; Rosenblatt, H.M.; Filipovich, A.H.; Adelstein, S.; Modi, W.S.; McBride, O.W.; Leonard, W.J. Interleukin-2 receptor gamma chain mutation results in X-linked severe combined immunodeficiency in humans. *Cell* **1993**, *73*, 147–157.

184. Buckley, R.H.; Schiff, R.I.; Schiff, S.E.; Markert, M.L.; Williams, L.W.; Harville, T.O.; Roberts, J.L.; Puck, J.M. Human severe combined immunodeficiency: Genetic, phenotypic, and functional diversity in one hundred eight infants. *J. Pediatr.* **1997**, *130*, 378–387.

185. Elsaesser, H.; Sauer, K.; Brooks, D.G. IL-21 is required to control chronic viral infection. *Science* **2009**, *324*, 1569–1572.

186. Spolski, R.; Leonard, W.J. Interleukin-21: Basic biology and implications for cancer and autoimmunity. *Annu. Rev. Immunol.* **2008**, *26*, 57–79.

187. Yi, J.S.; Du, M.; Zajac, A.J. A vital role for interleukin-21 in the control of a chronic viral infection. *Science* **2009**, *324*, 1572–1576.

188. Kotlarz, D.; Ziętara, N.; Milner, J.D.; Klein, C. Human IL-21 and IL-21R deficiencies: Two novel entities of primary immunodeficiency. *Curr. Opin. Pediatr.* **2014**, *26*, 704–712.

189. Salzer, E.; Kansu, A.; Sic, H.; Májek, P.; Ikincioğullari, A.; Dogu, F.E.; Prengemann, N.K.; Santos-Valente, E.; Pickl, W.F.; Bilic, I.; Ban, S.A.; Kuloğlu, Z.; Demir, A.M.; Ensari, A.; Colinge, J.; Rizzi, M.; Eibel, H.; Boztug, K. Early-onset inflammatory bowel disease and common variable immunodeficiency-like disease caused by IL-21 deficiency. *J. Allergy Clin. Immunol.* **2014**, *133*, 1651–1659.e12.

190. Márquez, A.; Orozco, G.; Martínez, A.; Palomino-Morales, R.; Fernández-Arquero, M.; Mendoza, J.L.; Taxonera, C.; Díaz-Rubio, M.; Gómez-García, M.; Nieto, A.; *et al*. Novel association of the interleukin 2-interleukin 21 region with inflammatory bowel disease. *Am. J. Gastroenterol.* **2009**, *104*, 1968–1975.

191. van Heel, D.A.; Franke, L.; Hunt, K.A.; Gwilliam, R.; Zhernakova, A.; Inouye, M.; Wapenaar, M.C.; Barnardo, M.C.N.M.; Bethel, G.; Holmes, G.K.T.; *et al*. A genome-wide association study for celiac disease identifies risk variants in the region harboring IL2 and IL21. *Nat. Genet.* **2007**, *39*, 827–829.

192. Liu, Y.; Helms, C.; Liao, W.; Zaba, L.C.; Duan, S.; Gardner, J.; Wise, C.; Miner, A.; Malloy, M.J.; Pullinger, C.R.; *et al*. A genome-wide association study of psoriasis and psoriatic arthritis identifies new disease loci. *PLoS Genet.* **2008**, *4*, e1000041.

193. Simpson, N.; Gatenby, P.A.; Wilson, A.; Malik, S.; Fulcher, D.A.; Tangye, S.G.; Manku, H.; Vyse, T.J.; Roncador, G.; Huttley, G.A.; *et al*. Expansion of circulating T cells resembling follicular helper T cells is a fixed phenotype that identifies a subset of severe systemic lupus erythematosus. *Arthritis Rheum.* **2010**, *62*, 234–244.

194. McPhee, C.G.; Bubier, J.A.; Sproule, T.J.; Park, G.; Steinbuck, M.P.; Schott, W.H.; Christianson, G.J.; Morse, H.C.; Roopenian, D.C. IL-21 is a double-edged sword in the systemic lupus erythematosus-like disease of BXSB.Yaa mice. *J. Immunol. Baltim. Md 1950* **2013**, *191*, 4581–4588.

195. Izui, S.; Ibnou-Zekri, N.; Fossati-Jimack, L.; Iwamoto, M. Lessons from BXSB and related mouse models. *Int. Rev. Immunol.* **2000**, *19*, 447–472.

196. Herber, D.; Brown, T.P.; Liang, S.; Young, D.A.; Collins, M.; Dunussi-Joannopoulos, K. IL-21 has a pathogenic role in a lupus-prone mouse model and its blockade with IL-21R.Fc reduces disease progression. *J. Immunol. Baltim. Md 1950* **2007**, *178*, 3822–3830.

197. McGuire, H.M.; Walters, S.; Vogelzang, A.; Lee, C.M.Y.; Webster, K.E.; Sprent, J.; Christ, D.; Grey, S.; King, C. Interleukin-21 is critically required in autoimmune and allogeneic responses to islet tissue in murine models. *Diabetes* **2011**, *60*, 867–875.

198. Kwok, S.-K.; Cho, M.-L.; Park, M.-K.; Oh, H.-J.; Park, J.-S.; Her, Y.-M.; Lee, S.-Y.; Youn, J.; Ju, J.H.; Park, K.S.; *et al*. Interleukin-21 promotes osteoclastogenesis in humans with rheumatoid arthritis and in mice with collagen-induced arthritis. *Arthritis Rheum.* **2012**, *64*, 740–751.

199. Erb, K.J.; Rüger, B.; von Brevern, M.; Ryffel, B.; Schimpl, A.; Rivett, K. Constitutive expression of interleukin (IL)-4 *in vivo* causes autoimmune-type disorders in mice. *J. Exp. Med.* **1997**, *185*, 329–339.

200. Singh, R.R. IL-4 and many roads to lupuslike autoimmunity. *Clin. Immunol. Orlando Fla* **2003**, *108*, 73–79.

201. Ma, J.; Zhu, C.; Ma, B.; Tian, J.; Baidoo, S.E.; Mao, C.; Wu, W.; Chen, J.; Tong, J.; Yang, M.; *et al*. Increased frequency of circulating follicular helper T cells in patients with rheumatoid arthritis. *Clin. Dev. Immunol.* **2012**, *2012*, 827480.

202. Zivojinovic, S.; Pejnovic, N.; Sefik-Bukilica, M.; Kovacevic, L.; Soldatovic, I.; Bugarski, D.; Mojsilovic, S.; Damjanov, N. Effects of TNF inhibitor on innate inflammatory and Th17 cytokines in stimulated whole blood from rheumatoid arthritis patients. *Inflammopharmacology* **2012**, *20*, 323–330.

203. Jang, E.; Cho, S.-H.; Park, H.; Paik, D.-J.; Kim, J.M.; Youn, J. A positive feedback loop of IL-21 signaling provoked by homeostatic CD4+CD25- T cell expansion is essential for the development of arthritis in autoimmune K/BxN mice. *J. Immunol. Baltim. Md 1950* **2009**, *182*, 4649–4656.

204. Nishimoto, N.; Kishimoto, T. Inhibition of IL-6 for the treatment of inflammatory diseases. *Curr. Opin. Pharmacol.* **2004**, *4*, 386–391. 

The authors would like to apologize for any inconvenience caused. The manuscript will be updated and the original will remain available on the article webpage.

## References

[1] Jandl C., King C. (2016). Cytokines in the Germinal Center Niche. Antibodies.

